# Removal models accounting for temporary emigration

**DOI:** 10.1111/biom.12961

**Published:** 2018-09-19

**Authors:** Ming Zhou, Rachel S. McCrea, Eleni Matechou, Diana J. Cole, Richard A. Griffiths

**Affiliations:** ^1^ School of Mathematics, Statistics and Actuarial Science University of Kent Canterbury U.K.; ^2^ Durrell Institute of Conservation and Ecology University of Kent Canterbury U.K.

**Keywords:** Abundance, Constraints, Hidden Markov models, Integrated modelling, Parameter redundancy, Robust design

## Abstract

Removal of protected species from sites scheduled for development is often a legal requirement in order to minimize the loss of biodiversity. The assumption of closure in the classic removal model will be violated if individuals become temporarily undetectable, a phenomenon commonly exhibited by reptiles and amphibians. Temporary emigration can be modeled using a multievent framework with a partial hidden process, where the underlying state process describes the movement pattern of animals between the survey area and an area outside of the study. We present a multievent removal model within a robust design framework which allows for individuals becoming temporarily unavailable for detection. We demonstrate how to investigate parameter redundancy in the model. Results suggest the use of the robust design and certain forms of constraints overcome issues of parameter redundancy. We show which combinations of parameters are estimable when the robust design reduces to a single secondary capture occasion within each primary sampling period. Additionally, we explore the benefit of the robust design on the precision of parameters using simulation. We demonstrate that the use of the robust design is highly recommended when sampling removal data. We apply our model to removal data of common lizards, *Zootoca vivipara*, and for this application precision of parameter estimates is further improved using an integrated model.

## 1 Introduction

Removal sampling is commonly used to estimate abundance of exploited populations in which captured individuals are permanently removed from a study area (Otis et al., 1978; Pollock, 1991; Hilborn and Walters, 1992). In recent years, it has been adopted as conservation management tools such as mitigation translocations (Germano et al., 2015) and removal of invasive species (Davis et al., 2016). We present our work in the context of mitigation translocations however it may be also useful for modeling invasive species removals.

Conservation mitigation translocations, the movement of protected species to prevent their extinction prior to the development of land, have become popular. Translocations involve capture, relocation and release or introduction of species from one area to another habitat. Amphibians and reptiles are frequently found at development sites in the United Kingdom, resulting in hundreds of such translocation projects annually (Germano et al., 2015). Although millions of pounds have been spent on removing protected animals out of the path of land development annually in the UK, such translocations may not meet the objective of preserving the target population as intended by legislation, and numerous reviews mention poor rates of success from these projects (e.g., Linnell et al., 1997). Failure of such projects can be a result of insufficient survey effort, resulting in too few animals being captured to establish a viable population elsewhere. Equally, many animals may go undetected at the removal site, resulting in the loss of the majority of the population when the site is developed. This raises questions concerning the amount of survey effort required to remove a significant proportion of the population. However, few publications highlight the need for improved statistical removal models to evaluate conservation actions (Griffiths et al., 2015). From a statistical perspective, the current abundance modeling approaches for removal data may give rise to misleading conclusions, as imperfect availability of individuals is ignored.

The work of this article is motivated by removal data sets which consist of counts recording the number of individuals removed at each occasion. The classic removal model was introduced by Moran (1951) and Zippin (1956) and relied on the assumptions of population closure and constant detection probability, meaning that all animals are assumed to be available for capture with the same probability throughout the study and there are no births, deaths or permanent immigrations/emigrations during the study. The basic removal model results in a geometric decline in the expected number of captured individuals over time. This classic removal model is a special case of model $M_{b}$ for closed populations which allows for a behavioral response to initial trapping (Otis et al., 1978).

Recently, removal models have been presented as a class of hierarchical models. Dorazio and Howard (2005) present a hierarchical removal model where the sites are assumed to have several distinct sub‐sites located spatially. Chandler et al. (2011) developed a spatially explicit temporary emigration model permitting the estimation of population density for point count data such as removal sampling, double‐observer sampling, and distance sampling. However, their model cannot be applied to removal data when spatial information is unavailable. More recently, Matechou et al. (2016) developed a Bayesian approach for removal data observed at a single site which allows for population renewal through birth/immigration as well as for population depletion through death/emigration in addition to the removal process. However, they assume that any emigration from the population is permanent.

Amphibians and reptiles exhibit temporary emigration as they have relatively limited dispersal abilities (e.g., up to a few hundred square metres for common lizards) and their daily activities are secretive. For instance, they can temporarily hide in shelters such as burrows and vegetation for extended periods resulting in zero detection probabilities during some sampling occasions for part of the population (Edgar et al., 2010). Their behavior is also highly weather dependent because they rely on the external environment to raise their body temperature. For example, slow‐worms, *Anguis fragilis*, a legless lizard, primarily live underground or underneath objects lying on the ground and although they may be detected basking on the ground to maintain their temperatures, most activity takes place out of sight of ecologists as they are fossorial (Edgar et al., 2010). Such temporary emigration can be modeled as a partial hidden process between two states that describes the underlying movement pattern of individuals between the study area and an area outside of the study. The multievent framework, formulated by Pradel (2005), accommodates state uncertainty for capture–recapture data. However, no approach currently exists for modeling temporary emigration for removal studies at a single study site. Ignoring such ecological features of species results in a positively biased estimate of the number of individuals left behind after the end of removal projects.

Pollock (1982) introduced the robust design and Schwarz and Stobo (1997) generalized the model in Pollock (1982) by allowing individuals to enter and leave the population between secondary samples. The robust design accommodates multiple secondary sampling occasions within each primary sampling period, and enables the estimation of temporary emigration from the study site (Kendall et al., 1995; Kendall and Bjorkland, 2001). The population is assumed to be open only for temporary emigration between primary occasions and closed within each primary sampling period. Such emigration can be modeled as a first‐order Markov process with different transition probabilities for individuals depending on which state they currently reside. Gould and Pollock (1997) first implemented a catch‐effort removal model under the robust design where the population is assumed to be open for survival and/or recruitment between primary periods.

Motivated by real data and ecological features of amphibians and reptiles, we develop novel removal models that bring together both the robust design and a multievent structure for removal data using maximum likelihood inference. The objective is to provide an unbiased estimate of the number of animals remaining at the site at the end of the removal project. The article is structured as follows: in Section 2, we describe the robust design multievent removal model (RMER) framework and the integrated RMER (IRMER) for modeling multiple populations simultaneously. Because of the complexity of the proposed models, it is not possible to estimate all of the model parameters in some cases; this is known as parameter redundancy (Cole et al., 2010). Parameter redundancy of the proposed RMER is explored in Section 3. Section 4 presents simulations for the proposed removal models and explores the benefits of the new modeling approach. Within section 5, we present the results obtained from fitting IRMER models to juvenile and adult data of common lizards, *Zootoca vivipara*. The article concludes with a discussion in Section 6.

## 2 Robust Design Multi‐Event Removal Model

### 2.1 Notation

Consider a removal experiment conducted at a site with a population of *N* individuals. *N* is the total number of animals that become exposed to sampling efforts at least once during the study. Individuals are permanently removed from the study area once captured during the study period. Suppose, there are two states in the model: individuals in state 1 are present and available for removal, while individuals in state 2 are absent from the study site and hence unavailable for capture. We assume the removal study is conducted within a robust design framework which comprises of primary periods i=1,…,T and secondary sampling occasions $j=1,\ldots ,k_{i}$ within the *i*th primary period. The population is assumed to be open for temporary emigration only (i.e., no recruitment or permanent departures) between primary periods and we assume population closure between secondary samples within a primary session. The total number of sampling occasions is denoted by $K(=\sum_{i=1}^{T}k_{i})$. The removal data that arise is an array with entry $n_{i,j}$ representing the number of individuals removed at the *j*th secondary occasion within the *i*th primary period. The total number of individuals removed is denoted by $D(=\sum_{i=1}^{T}\sum_{j=1}^{k_{i}}n_{i,j}$). We define:

- $n_{0}$: the number of animals that have not been removed by the end of the study, where $n_{0}=N-D$.
- π: the initial state matrix, defined as a row vector, (π,1−π), where π represents the proportion of individuals in state 1 and the complement of π, 1−π, the proportion of individuals in state 2, at the start of the study.
- $\Phi_{i}$: the state transition matrix, where $\varphi_{i}^{12}$ and $\varphi_{i}^{21}$ are transition probabilities from state 1 to state 2, and transition probabilities from state 2 to state 1, respectively, between the *i*th and (i+1)th primary period, where i=1,…,T−1.
  $$\Phi_{i}=\left(\begin{array}{cc} 1-\varphi_{i}^{12} & \varphi_{i}^{12}\\ \varphi_{i}^{21} & 1-\varphi_{i}^{21} \end{array}\right)$$
- $B_{i,j}$: the state‐event matrix, where events are “Removed” and “Not Removed” in the first and second column, respectively. States 1 and 2 are in the first and second row, respectively. $p_{i,j}$ is the probability that an individual is captured at the *j*th secondary sample within the *i*th primary period. The detection probability matrix (denoted as $P_{i,j}$) is a diagonal matrix with elements equal to the first column vector of $B_{i,j}$. Similarly, $Q_{i,j}$ is the diagonal probability matrix of not detecting individuals.
  $$B_{i,j}=\left(\begin{array}{cc} p_{i,j} & 1-p_{i,j}\\ 0 & 1 \end{array}\right)$$
  $$P_{i,j}\;=\;D\{B_{i,j}(\cdot ,1)\}\;=\;\left(\begin{array}{cc} p_{i,j} & 0\\ 0 & 0 \end{array}\right),Q_{i,j}\;=\;D\{B_{i,j}(\cdot ,2)\}\;=\;\left(\begin{array}{cc} 1-p_{i,j} & 0\\ 0 & 1 \end{array}\right)$$

Constant parameters are designated by the absence of a subscript from the corresponding time‐specific parameters, for example, *p* denotes a constant capture probability over time.

### 2.2 The Likelihood Formulation

We adopt a multievent approach taking into account the robust design framework for the computation of the likelihood function. The probability of an individual being removed at the *j*th sample within the first primary period is, $L_{1,j}=\pi Q_{1,0}Q_{1,1}\ldots Q_{1,j-1}P_{1,j}1_{2}$, where $j=1,\ldots ,k_{1}$, $Q_{1,0}=I_{2}$ is the 2×2 identity matrix and $1_{2}$ is the column vector of two ones (and thereafter). The probability of an individual being removed at the *j*th sample within the *i*th primary period is,

$$L_{i,j}=\pi Q_{1,0}\ldots Q_{1,k_{1}}\Phi_{1}Q_{2,0}\ldots Q_{2,k_{2}}\Phi_{2}\ldots Q_{i,k_{i}-1}P_{i,j}1_{2}$$

where i=2,…,T, $j=1,\ldots ,k_{i}$ and $Q_{i,0}=I_{2}$ is the 2×2 identity matrix.

The probability of not being removed by the end of the study is given by

$$L_{0}=\pi Q_{1,0}\ldots Q_{1,k_{1}}\Phi_{1}Q_{2,0}\ldots Q_{2,k_{2}}\Phi_{2}\ldots Q_{T,k_{T}-1}Q_{T,k_{T}}1_{2}.$$

The full product multinomial likelihood is given by

(1) $$L(\pi ,\varphi_{i}^{12},\varphi_{i}^{21},p_{i,j},n_{0})=\frac{N!}{n_{0}!\prod_{i=1}^{T}\prod_{j=1}^{k_{i}}n_{k}!}L_{0}^{n_{0}}\prod_{i=1}^{T}\prod_{j=1}^{k_{i}}L_{i,j}^{n_{k}}.$$

We note that the likelihood function (1) can be easily adapted to accommodate multiple species or different age/sex groups for a single species using an integrated population modeling approach (Chapter 12 Besbeas et al., 2002; McCrea and Morgan, 2014). Consider a removal experiment conducted on *W* species (or *W* categories for a single species) with the individual likelihoods defined as $L_{1},L_{2},\ldots ,L_{W}$. Assuming the groups of individuals are removed independently, the full likelihood *L* can be written as the product of individual likelihoods, that is, $L=L_{1}\times L_{2}\times \ldots \times L_{W}$.

The model belongs to the family of hidden Markov models (Pradel, 2005; Zucchini et al., 2016), so standard errors can be obtained from the Hessian. When some parameters lie on the boundary, non‐parametric bootstrap can be used instead to compute standard errors and confidence intervals (Section 3.6 Zucchini et al., 2016).

### 2.3 Constraints

The model that assumes fully time‐dependent parameters (π, $p_{i,j}$, $\varphi_{i}^{12}$, $\varphi_{i}^{21}$, $n_{0}$) has K+2T parameters and the number of observations is *K*. This model is parameter redundant (Cole et al., 2010) because it has more parameters than the number of observed data points. Therefore, we cannot estimate all of the parameters individually without further constraints. The use of constraints has been suggested in the literature (e.g., Kendall et al., 1997; Williams et al., 2002). One natural way of enabling the estimation of parameters involves constraining them to be constant over time. However, capture probability is rarely constant at different sampling occasions and a closed removal model requires covariates to estimate time‐varying capture probability (Williams et al., 2002). Kendall et al. (1997) recommend to constrain the last two transitions to be equal in order to provide identifiability of the last survival parameter in a time‐varying temporary emigration model. We describe a list of constraints for our proposed models below:

- Constraint related to detection probability: The time‐dependent capture probability $p_{i,j}$ can be modeled using a logistic regression in terms of covariate $z_{i,j}$ at the *j*th secondary occasion within the *i*th primary period, that is, $\mathrm{logit}(p_{i,j})=\log\{p_{i,j}/(1-p_{i,j})\}=\alpha +\beta z_{i,j}$ (Madsen and Thyregod, 2011). We label this constraint “Z”.
- Constraint related to initial state parameter: $\pi =\{1/(T-1)\}\sum_{i=1}^{T-1}\{\varphi_{i}^{21}/(\varphi_{i}^{12}+\varphi_{i}^{21})\}$, the initial state parameter π can be constrained using this expression if we assume the population is initially allocated to two states according to the mean of the stationary distributions of the transition matrices across time which is $\left\{\varphi_{i}^{21}/(\varphi_{i}^{12}+\varphi_{i}^{21}),\varphi_{i}^{12}/(\varphi_{i}^{21}+\varphi_{i}^{21})\right\}$, i=1,…,T−1. We label this constraint “S” to represent that the stationary distribution is being assumed for π.
- Constraint related to transition probability: The superscript “t in the following constraints denotes fully time‐dependent transition probabilities and the absence of the superscript indicates that constant transition parameters are assumed.
  ‐ $\varphi_{i}^{12}+\varphi_{i}^{21}=1$. This constraint is equivalent to the random emigration/movement model for capture recapture data sampled with robust design as described in Kendall et al. (1997). It suggests that the probability of being in the unobservable state between the *i*th and (i+1)th primary session is the same for individuals in and individuals outside the study area. In this case, we treat the transition probability $\varphi_{i}^{12}$ as a free parameter to be estimated in the model, and the transition probability $\varphi_{i}^{21}$ is reparameterized using the constraint, that is, $\varphi_{i}^{21}=1-\varphi_{i}^{12}$. This constraint is labeled “R” and “$R^{t}$” to denote constant and time‐dependant random emigration, respectively. Furthermore, we extend this constraint in Web Appendix C to $\varphi_{i}^{12}+\varphi_{i}^{21}=v$ for v∈(0,2) where *v* is an additional parameter.
  ‐ $\varphi_{i}^{12}=\varphi_{i}^{21}$. This is an “even flow” model (Kendall et al., 1997), where the probability of transitioning from the study area to an unobservable state is the same as the probability of moving back to the study area between the *i*th and (i+1)th primary period. This is denoted by “$E^{t}$” and “E” for time‐varying and constant transition parameters, respectively.
  ‐ $\varphi_{T}^{12}=\varphi_{T-1}^{12}$ and $\varphi_{T}^{21}=\varphi_{T-1}^{21}$; the penultimate and final transition probabilities are assumed to be equal, an approach commonly used to permit identifiability of the parameters in the first‐order Markovian robust design model for capture recapture data (Kendall et al., 1997). We denote this constraint by “2” as the last two transitions are assumed to be equal. If constraint “2” is combined with constraint “$R^{t}$” or “$E^{t}$”, “$R_{2}^{t}$” or “$E_{2}^{t}$” are used.
  ‐ Suppose, we have *W* populations of interest indexed as w=1,…,W. Time‐dependent transition probabilities for population *w*, for example $\varphi_{i,w}^{12}$, can be modeled using $\mathrm{logit}(\varphi_{i,w}^{12})=\log\{\varphi_{i,w}^{12}/(1-\varphi_{i,w}^{12})\}=\eta_{1,i}+\gamma_{w}$, where $\eta_{1,i}$ is the logit of transition probabilities for a baseline population (numbered 1) and $\gamma_{w}$ represents an additive effect of group *w*. The use of this constraint reduces the complexity of the model and enables better precision by sharing additional information across different groups. We denote this constraint by “$R_{a}^{t}$” or “$E_{a}^{t}$” to represent the additive effect for the time‐varying transition probabilities. If we have more than two populations, we can use “$R_{a,w}^{t}$” or “$E_{a,w}^{t}$” to represent the additive effect for $\varphi_{i,w}^{12}$ for population *w*. In addition, for the IRMER models the absence of the subscript in “$R_{a}^{t}$” and “$E_{a}^{t}$” (i.e., “$R^{t}$” and “$E^{t}$”) denotes that time‐dependent transition probabilities are equal for both of the populations accounting for the same constraint.

We propose a model name that is composed of both the model structure and the different combinations of constraints for all models that we consider. We employ the structure of “MODEL‐XYZ” in Tables 1, 2, and 3, where “X”, “Y” and “Z” respectively represent the constraint used for the initial state parameter (e.g., “S”), the transition probabilities (e.g., “$R_{a}^{t}$”) and whether the capture probability is constant over time (“C”) or time‐dependent in term of covariates (“Z”). In addition, “N” suggests that no constraint has been considered. “MODEL” is either “R” or “IR” for the RMER and IRMER models, respectively. For the MER models, we ignore the “MODEL‐” and only use “XYZ” to denote the model. For the IRMER models, we use the population number as the subscript in “S” to denote which population is subject to the constraint “S”, for example, “$S_{1,2}$” indicates that both initial state distributions for population 1 and 2 are assumed to be stationary. For clarity, ${\mathrm{IR}-\mathrm{NE}}_{2}$C denotes the IRMER model with no constraint for the initial state parameter, constraint “$E_{2}^{t}$” for the time‐varying transition probabilities and a constant capture probability.

## 3 Parameter Redundancy

A model is parameter redundant if it is impossible to estimate all the parameters individually, because the model could be reparameterised in terms of a small number of parameters (Catchpole and Morgan, 1997). The techniques for detecting parameter redundancy have been developed for a wide range of applications, (Catchpole and Morgan, 1997; Cole et al., 2010; Cole and McCrea, 2016).

We employ the methods of Cole et al. (2010) to assess whether our constrained RMER and IRMER models are parameter redundant by forming a derivative matrix D=∂κ(θ)/∂θ, where κ(θ) is a vector of parameter combinations that represents the structure of the model for a set of parameters θ. Once D is formed, we can determine whether or not the model is parameter redundant by calculating the rank of **D**, *r*. The deficiency of the model, *d*, is the number of parameters, *h*, minus *r*. If d>0 the model is parameter redundant, otherwise if d=0 the model is not parameter redundant, and termed full rank. We also show which combinations of parameters are estimable if the model is parameter redundant by solving a system of first‐order partial differentiation equations (Cole et al., 2010). This method is explained in full in Web Appendix A, which includes an illustrative example for the model R‐NNC. Software Maple has been used for the symbolic algebra computations.

We obtain general parameter redundancy results for our removal models with an even number of total sampling occasions with two secondary samples within each primary period in Tables 1 and 2. In addition to numbering all the models with model codes, we also denote different models by their constituent parameters. We show the estimable combinations of parameters for parameter redundant models in Table 1 only in the article and more results for Table 2 are available in Web Appendix A.

**Table 1:** Parameter redundancy results of RMER and MER models, where $\varphi^{12}$ and $\varphi^{21}$ are constant over time. The parameter redundancy results hold for *K* sampling occasions, where there are two secondary samples for each primary period and K≥5. *h* is the number of parameters in the model. *d* is the deficiency of the model. “t” denotes time‐dependence, “c” denotes a constant parameter, and “t+cov” denotes additive effect in terms of covariates. The PRS column represents the parameter redundancy status, where FR indicates that all parameters in the model are theoretically estimable, PR indicates that the model is parameter redundant, NR indicates that the model is full rank but near redundant for the scenarios we considered. ${}^{\#}$ indicates that the estimable combination of parameters are πp, $\varphi^{12}p$ and $(\varphi^{12}-1)p-\varphi^{12}-\varphi^{21}$. ${}^{†}$ indicates that the estimable combinations of parameters are πp and $(\varphi^{12}-1)p$. ${}^{‡}$ indicates that the estimable combination of parameters is $(\varphi^{12}-1)p$

		RMER			MER		
Model	*h*	Model code	*d*	PRS	Model code	*d*	PRS
$\pi ,\varphi^{12}(c),\varphi^{21}(c),p(c)$	4	R‐NNC	0	NR	NNC	1	PR,${}^{\#}$
$\pi ,\varphi^{12}(c),\varphi^{21}(c),p(t+\mathrm{cov})$	5	R‐NNZ	0	NR	NNZ	0	NR
$\varphi^{12}(c),\varphi^{21}(c),p(c)$	3	R‐SNC	0	FR	SNC	0	NR
$\pi ,\varphi^{12}(c),p(c)$	3	R‐NRC	0	FR	NRC	1	PR,${}^{†}$
$\pi ,\varphi^{12}(c),p(c)$	3	R‐NEC	0	FR	NEC	0	NR
$\varphi^{12}(c),\varphi^{21}(c),p(t+\mathrm{cov})$	4	R‐SNZ	0	FR	SNZ	0	NR
$\pi ,\varphi^{12}(c),p(t+\mathrm{cov})$	4	R‐NRZ	0	FR	NRZ	0	NR
$\pi ,\varphi^{12}(c),p(t+\mathrm{cov})$	4	R‐NEZ	0	FR	NEZ	0	NR
$\varphi^{12}(c),p(c)$	2	R‐SRC	0	FR	SRC	1	PR,${}^{‡}$
$\varphi^{12}(c),p(c)$	2	R‐SEC	0	FR	SEC	0	NR
$\varphi^{12}(c),p(t+\mathrm{cov})$	3	R‐SRZ	0	FR	SRZ	0	NR
$\varphi^{12}(c),p(t+\mathrm{cov})$	3	R‐SEZ	0	FR	SEZ	0	NR

**Table 2:** Parameter redundancy results for RMER models, where $\varphi_{i}^{12}$ and $\varphi_{i}^{21}$ are time‐varying over time. The parameter redundancy results hold for an even number of total sampling occasions *K*, where there are two secondary samples for each primary period and K≥8. *h* is the number of parameters in the model. *d* is the deficiency of the model. “t” denotes time‐dependence, “c” denotes a constant parameter, and “t+cov” denotes additive effect in terms of covariates. “$t+\gamma_{2}$” denotes additive effect for transition probabilities. PRS represents the parameter redundancy status, where FR indicates that all parameters in the model are theoretically estimable, PR indicates that the model is parameter redundant, NR indicates that the model is full rank but near redundant for the scenarios we considered. All results hold for K≥4 occasions, except that * indicates the results hold for K≥6 occasions and ** indicates the results hold for K≥8 occasions

Model code	Model	*h*	*d*	PRS
	RMER model			
${R-\mathrm{NN}}^{t}$C	$\pi ,\varphi^{12}(t),\varphi^{21}(t),p(c)$	*K*	K/2−1	PR
${R-\mathrm{NN}}^{t}$Z	$\pi ,\varphi^{12}(t),\varphi^{21}(t),p(t+\mathrm{cov})$	K+1	K/2−1	PR
${R-\mathrm{SN}}^{t}$C	$\varphi^{12}(t),\varphi^{21}(t),p(c)$	K−1	K/2−2	PR
${R-\mathrm{NR}}^{t}$C	$\pi ,\varphi^{12}(t),p(c)$	K/2+1	0	NR
${R-\mathrm{NE}}^{t}$C	$\pi ,\varphi^{12}(t),p(c)$	K/2+1	0	NR
${R-N2}^{t}$C	$\pi ,\varphi^{12}(t),\varphi^{21}(t),p(c)$	K−2	K/2−3	PR *
${R-\mathrm{SN}}^{t}$Z	$\varphi^{12}(t),\varphi^{21}(t),p(t+\mathrm{cov})$	*K*	K/2−2	PR
${R-\mathrm{NR}}^{t}$Z	$\pi ,\varphi^{12}(t),p(t+\mathrm{cov})$	K/2+2	0	NR
${R-\mathrm{NE}}^{t}$Z	$\pi ,\varphi^{12}(t),p(t+\mathrm{cov})$	K/2+2	0	NR
${R-N2}^{t}$Z	$\pi ,\varphi^{12}(t),\varphi^{21}(t),p(t+\mathrm{cov})$	K−1	K/2−3	PR *
${R-\mathrm{SR}}^{t}$C	$\varphi^{12}(t),p(c)$	K/2−1	0	FR
${R-\mathrm{SE}}^{t}$C	$\varphi^{12}(t),p(c)$	K/2−1	0	NR
${R-S2}^{t}$C	$\varphi^{12}(t),\varphi^{21}(t),p(c)$	K−3	K/2−4	PR **
${R-\mathrm{NR}}_{2}^{t}$ C	$\pi ,\varphi^{12}(t),p(c)$	K/2	0	NR
${R-\mathrm{NE}}_{2}^{t}$ C	$\pi ,\varphi^{12}(t),p(c)$	K/2	0	NR
${R-\mathrm{SR}}^{t}$Z	$\varphi^{12}(t),p(t+\mathrm{cov})$	K/2+1	0	FR
${R-\mathrm{SE}}^{t}$Z	$\varphi^{12}(t),p(t+\mathrm{cov})$	K/2+1	0	NR
${R-S2}^{t}$ Z	$\varphi^{12}(t),\varphi^{21}(t),p(t+\mathrm{cov})$	K−2	K/2−4	PR **
${R-\mathrm{NR}}_{2}^{t}$ Z	$\pi ,\varphi^{12}(t),p(t+\mathrm{cov})$	K/2+1	0	NR
${R-\mathrm{NE}}_{2}^{t}$ Z	$\pi ,\varphi^{12}(t),p(t+\mathrm{cov})$	K/2+1	0	NR
${R-\mathrm{SR}}_{2}^{t}$ C	$\varphi^{12}(t),p(c)$	K/2−1	0	FR
${R-\mathrm{SR}}_{2}^{t}$ Z	$\varphi^{12}(t),p(t+\mathrm{cov})$	K/2	0	FR
	IRMER model			
${\mathrm{IR}-\mathrm{NR}}^{t}$C	$\pi_{1},\pi_{2},\varphi^{12}(t),p(c)$	K/2+2	0	NR
${\mathrm{IR}-S}_{1,2}R^{t}$C	$\varphi^{12}(t),p(c)$	K/2	0	FR
${\mathrm{IR}-S}_{1,2}R_{a}^{t}C$	$\varphi^{12}(t),\varphi^{12}(t+\gamma_{2}),p(c)$	K/2+1	0	FR
${\mathrm{IR}-S}_{1}R_{a}^{t}C$	$\pi_{2},\varphi^{12}(t),\varphi^{12}(t+\gamma_{2}),p(c)$	K/2+2	0	FR

Catchpole et al. (2001) found that a full rank model can perform badly in practice, because the model is close to a nested parameter redundant model; this is known as near redundancy. In a parameter redundant model the expected information matrix will be singular (Rothenberg, 1971). As a result, the expected information matrix will have at least one zero eigenvalue. In a near redundant model, the smallest eigenvalue will be close to zero rather than exactly zero (Catchpole et al., 2001). Hence, even some of the full rank models in Tables 1 and 2 can give biased results regardless of how large the sample sizes are (see Web Appendix A). We list the parameter redundancy status for each model in Tables 1 and 2 to indicate whether the model is parameter redundant, full rank or near redundant.

Considering the results in Table 1, the specified RMER models are full rank for all cases. As a result, the robust design improves the estimation of the models in general, as the secondary samples within each primary period provide an additional source of information about capture probability. We also observe that the MER models are either parameter redundant or near redundant. Table 2 shows that all models with both fully time‐dependent transition probabilities $\varphi_{i}^{12}$ and $\varphi_{i}^{21}$ (i.e., without any constraint for $\varphi_{i}^{12}$ and $\varphi_{i}^{21}$) are parameter redundant. ${R-\mathrm{NN}}^{t}$C becomes full rank with constraint “$R^{t}$”. Furthermore, the issue of near redundancy for ${R-\mathrm{NR}}^{t}$C is overcome if constraint “S” is used for the initial state parameter.

We also investigate the parameter redundancy of IRMER models using two populations (numbered 1 and 2) with results shown in the last rows of Table 2. All of these IRMER models are determined to be full rank. However, we find ${\mathrm{IR}-\mathrm{NR}}^{t}$C is near redundant. Hence, we conclude that we need to apply at least constraints “S” and “$R^{t}$” in order to avoid parameter redundant and near redundant models.

## 4 Simulation Results

The aim of these simulations is to examine the precision of maximum likelihood estimators for RMER, MER, and IRMER within the likely range of ecological applications of the model. Three simulation settings are investigated, where RMER and MER models under *Setting 4.1* have constant transition probabilities, RMER models under *Setting 4.2* have time‐varing transition probabilities and *Setting 4.3* presents results obtained from IRMER models. For *Setting 4.1* and *4.2*, 500 simulations are conducted for a study with N=500 individuals, K=10 or K=20, with T=5 or T=10 primary periods and 2 secondary sampling occasions within each primary period (i.e., $k_{1}=\ldots =k_{T}=2$). For *Setting 4.3*, we conduct simulations for IRMER, where 500 replicates are simulated for a removal study with two populations (numbered 1 and 2) where the population sizes are N=300 and M=200. We consider eight scenarios because the performance of the models depends on the relationship between $\varphi_{i}^{12}$ and $\varphi_{i}^{21}$ and on capture probability. We only show the simulation results under Scenarios 1 and 2 in the article. More simulations are available in Web Appendix B. The true values of parameters used in the simulations are presented in the subsequent sections.

Scenario 1: low capture probability and individuals tend to stay offsite,
Scenario 2: low capture probability and individuals tend to stay onsite.

### Setting 4.1 RMER/MER with Constant Transition Probabilities

We only show results from R‐NNC, R‐SNC, R‐NRC, R‐SRC, and SRC in Table 1. We are interested in the precision of the estimators for the constraints used/not used for the initial state parameter and the transition probability for the RMER models. Furthermore, we demonstrate the distribution of the estimates for R‐NNC, which is classified as a near redundant model in Table 1. In addition, we show the results for the SRC model where both “S” and “R” are taken into account but without the robust design for comparison.

The true value of the constant capture probability is 0.3 under both Scenarios 1 and 2. In addition, we use $\varphi^{12}=0.8$, $\varphi^{21}=0.2$ in Scenario 1, when individuals tend to move to the unobservable state, while in Scenario 2, we define $\varphi^{12}=0.4$, $\varphi^{21}=0.6$ so that individuals tend to move to the observable state. The true value of the initial state parameter π is defined as the first element of the stationary distribution of the corresponding transition matrix.

**Figure 1:**
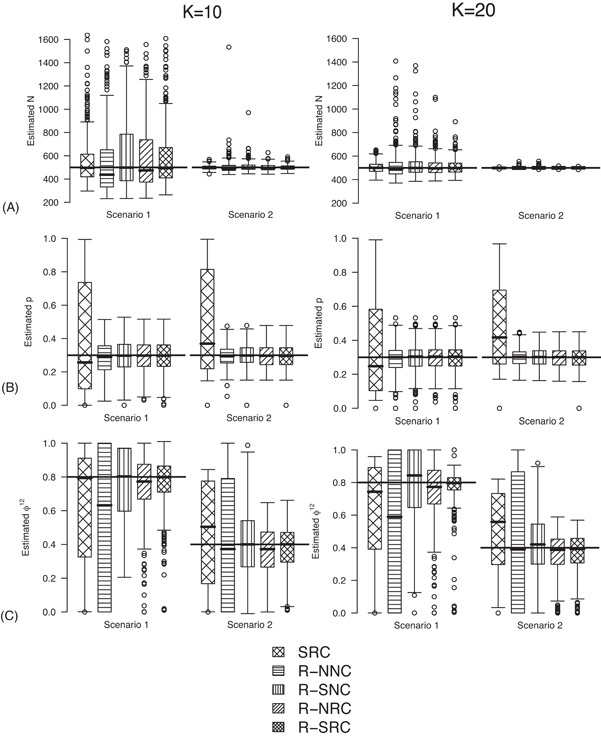
Estimated population size *N* (A), capture probability *p* (B) and transition probability $\varphi^{12}$ (C) for simulations with K=10 and K=20 sampling occasions under simulation *Setting 4.1*. The black horizontal lines are the true values used for simulation.

As shown in Figure 1, it is clear that estimation of population size *N* is reliable for all models when K=20, although long positive tails are recognized under Scenario 1 when individuals tend to emigrate offsite and capturing them becomes impossible. When K=10, longer positive tails are observed and the estimates of population size are negatively biased for models R‐NNC and R‐NRC under Scenario 1. The results for detection probability *p* show that the use of the robust design considerably improves the performance in terms of bias, compared with the SRC model that exhibits large bias for *p* in all cases. The bias in estimating $\varphi_{12}$ is modest for R‐SRC with both constraints “S” and “R” for all cases even when K=10. In contrast, estimation of $\varphi_{12}$ for SRC without the robust design is not reliable for any cases. In addition, R‐NNC yields biased estimates for $\varphi^{12}$ due to near redundancy. Overall, we conclude that R‐SRC performs the best as shown in Figure 1.

### Setting 4.2 RMER with Time‐Varying Transition Probabilities

RMER models with constant transition probabilities may not be realistic for real data as individuals may tend to stay in one state at times. Here we investigate the full‐rank models ${R-\mathrm{NR}}^{t}$C, ${R-\mathrm{SR}}^{t}$C, ${R-\mathrm{SR}}_{2}^{t}$C in Table 2 by simulation.

The true value of constant capture probability *p* is 0.3 for both Scenarios 1 and 2. In addition, the true transition probabilities $\varphi_{i}^{12}$ for simulating the data for RMER models under Scenario 1 when K=20 are (0.8, 0.7, 0.8, 0.3, 0.6, 0.7, 0.8, 0.6, 0.6) where individuals tend to stay in the area outside the study for the majority of times which is more realistic for real data. Furthermore, the vector of true $\varphi_{i}^{12}$ is defined as (0.4, 0.4, 0.8, 0.4, 0.4, 0.8, 0.4, 0.4, 0.4) under Scenario 2. For a study with K=10 occasions, we specify the true $\varphi_{i}^{12}$ as (0.7, 0.2, 0.7, 0.7) for Scenario 1, and (0.3, 0.8, 0.3, 0.3) for Scenario 2. We only display the estimates of $\varphi_{i}^{12}$ for the first two and the last two transitions in the article. The value of the initial state parameter π is set to be the mean of the first element of the stationary distributions of transition matrices across time.

**Figure 2:**
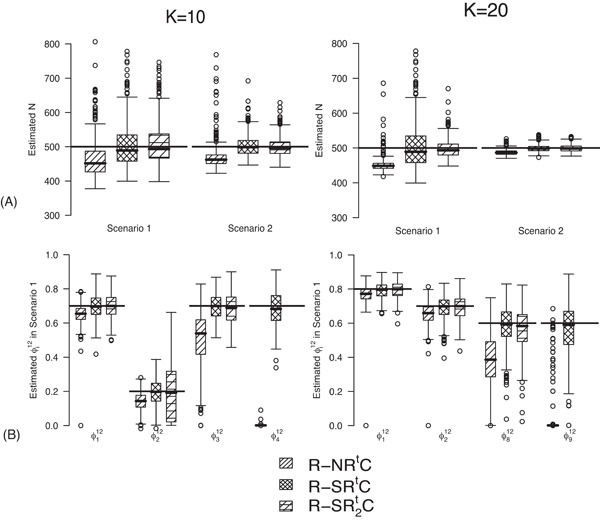
Simulation results of *Setting 4.2* with K=10 and K=20 sampling occasions. (A) Estimates of population size *N* under Scenarios 1 and 2, (B) estimates of transition probabilities for the first two and last two transitions under Scenario 1. The black horizontal lines are the true values used for simulation.

The bias in the estimation of *N* is negative for ${R-\mathrm{NR}}^{t}$C. The estimates of *N* are slightly biased low for ${R-\mathrm{SR}}^{t}$C under Scenario 1 and unbiased for other Scenarios (See Figure 2 and Web Appendix B). The results of the estimated transition probabilities in Figure 2 suggest unbiased estimates obtained from ${R-\mathrm{SR}}^{t}$C and negative bias from ${R-\mathrm{NR}}^{t}$C. This is expected as R‐SRC with constant $\varphi^{12}$ performs better than R‐NRC under *Setting 4.1*. The performance of ${R-\mathrm{SR}}^{t}$C with time‐varying $\varphi_{i}^{12}$ is reliable for real data in practice. None of the remaining RMER models in Table 2 yields unbiased estimates of time‐dependent transition probabilities apart from ${R-\mathrm{SR}}_{2}^{t}$C. We observe biased estimates for the RMER models with constraint “$E^{t}$” due to near redundancy (see more simulation results in Web Appendix B). Therefore, we conclude that good performance can be obtained for the RMER models with time‐varying $\varphi_{i}^{12}$ with at least the combination of constraints “S” and “$R^{t}$”.

### Setting 4.3 IRMER with Time‐Varying Transition Probabilities

Under this setting, we investigate the IRMER models in Table 2. We only show the results obtained for population size for ${\mathrm{IR}-S}_{1,2}R_{a}^{t}$C and ${\mathrm{IR}-S}_{1}R_{a}^{t}$C in the article. The true values of parameters for population 1 are the same as those under *Setting 4.2*. We define the true value of the additive effect $\gamma_{2}$ for population 2 to be −0.5 when constraint “$R_{a}^{t}$” is used.

**Figure 3:**
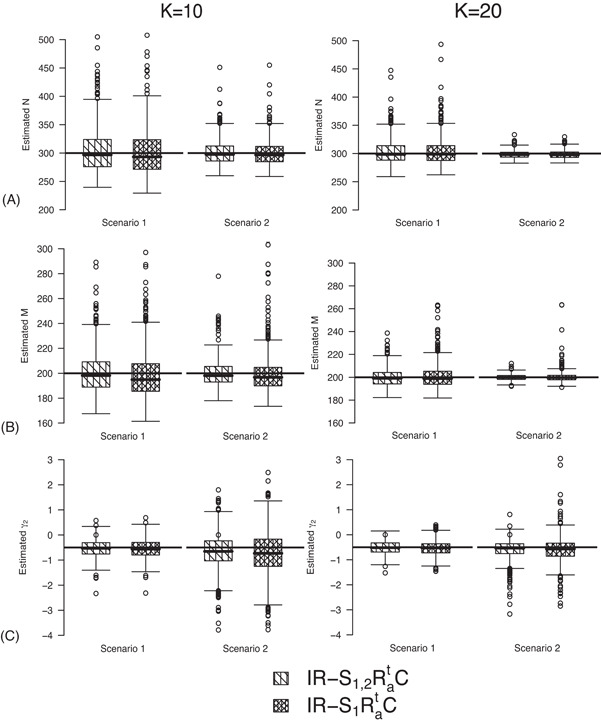
Estimated population sizes *N* (A), *M* (B) and additive effect $\gamma_{2}$ on transition probabilities (C) for simulations under *Setting 4.3* with K=10 and K=20 sampling occasions. The black horizontal lines are the true values used for simulating the data.

The estimates of population size for two populations and the additive effect $\gamma_{2}$ obtained from IRMER modeling are displayed in Figure 3. Estimation is unbiased for K=20 sampling occasions, while ${\mathrm{IR}-S}_{1}R_{a}^{t}$C slightly underestimates population sizes under Scenario 1 when K=10. Moreover, estimation of $\gamma_{2}$ is unbiased under Scenario 1 for both K=10 and K=20. However, when we have a small number of sampling occasions (K=10) under Scenario 2, $\gamma_{2}$ is slightly underestimated and estimation becomes unbiased for K=20.

We also show that the classic geometric removal model (denoted as GRM) overestimates the number of animals remaining at the study area at the end of the study and underestimate capture probability for the simulated data exhibiting temporary emigration with the robust design sampling protocol (Web Appendix B).

## 5 Application to Common Lizards

Removal of common lizards, *Zootoca vivipara*, was conducted daily in both the morning and afternoon from the September 13, 2010 to October 29, 2010. There were 94 sampling occasions, with 13 missed visits. 334 common lizards were captured and permanently removed from the study site. The removals consisted of 274 juvenile and 60 adult individuals. Eight covariates: mean/maximum/minimum air temperature, precipitation, average/maximum/minimum humidity and season stage, were recorded daily.

Migration and dispersal of reptiles and amphibians are generally limited during daytime (Edgar et al., 2010), therefore we used a robust design approach for our analysis, with days corresponding to primary periods and the repeated samples within days being the secondary sampling occasions. Hence, there are T=47 primary occasions and two secondary samples within each primary period. Given the nature of the available data, we assume juveniles and adults are sampled independently and hypothesize that their transition probabilities may be related. This is ecologically sensible since the dynamics exhibited by the population are likely to be driven by external influences. We define the global likelihood to be the product of individual likelihoods, that is, $L=L_{\mathrm{ju}}L_{\mathrm{ad}}$ where $L_{\mathrm{ju}}$ and $L_{\mathrm{ad}}$ are the likelihood for juvenile and adult populations respectively, and are both of the form described in equation (1).

We also considered incorporating the climatic covariates using a logistic regression to account for the time variation exhibited within the transition and capture probabilities. The likelihood is maximized using the optimizer optim in R (R Core Team, 2017). The results from performing model selection on the integrated data are displayed in Table 3, where the 10 IRMER models with the lowest Akaike information criterion (AIC) values are shown.

**Table 3:** List of models fitted to common lizard data. *h* is the number of parameters in the model, ML is the value of the maximized loglikelihood, ΔAIC are computed as the difference in the AIC value between the current and the best model, where AIC is the Akaike information criterion calculated as −2ML+2h. Only the 10 models with lowest AIC are shown. “t” denotes time‐dependence, “c” denotes a constant parameter, and “t+cov” denotes additive effect in terms of covariates

Model code	Model	Covariate	*h*	ML	ΔAIC
	IRMER model				
${\mathrm{IR}-S}_{\mathrm{ju},\mathrm{ad}}R_{a}^{t}Z$	$\varphi_{\mathrm{ju}}^{12}(t),\varphi_{\mathrm{ad}}^{12}(t+\gamma_{\mathrm{ad}}),p(t+\mathrm{cov})$	Precipitation	51	−259.53	0
${\mathrm{IR}-S}_{\mathrm{ju}}R_{a}^{t}Z$	$\pi_{\mathrm{ad}},\varphi_{\mathrm{ju}}^{12}(t),\varphi_{\mathrm{ad}}^{12}(t+\gamma_{\mathrm{ad}}),p(t+\mathrm{cov})$	Precipitation	52	−259.85	2.64
${\mathrm{IR}-S}_{\mathrm{ju},\mathrm{ad}}R^{t}Z$	$\varphi_{\mathrm{ju}}^{12}(t),p(t+\mathrm{cov})$	Precipitation	50	−261.94	2.82
${\mathrm{IR}-S}_{\mathrm{ju}}R_{a}^{t}Z$	$\pi_{\mathrm{ad}},\varphi_{\mathrm{ju}}^{12}(t),\varphi_{\mathrm{ad}}^{12}(t+\gamma_{\mathrm{ad}}),p(t+\mathrm{cov})$	Average humidity	52	−262.04	7.02
${\mathrm{IR}-S}_{\mathrm{ju},\mathrm{ad}}R_{a}^{t}C$	$\varphi_{\mathrm{ju}}^{12}(t),\varphi_{\mathrm{ad}}^{12}(t+\gamma_{\mathrm{ad}}),p(c)$	‐	50	−269.86	17.46
${\mathrm{IR}-S}_{\mathrm{ju},\mathrm{ad}}R_{a}^{t}Z$	$\varphi_{\mathrm{ju}}^{12}(t),\varphi_{\mathrm{ad}}^{12}(t+\gamma_{\mathrm{ad}}),p(t+\mathrm{cov})$	Mean air temperature	51	−268.98	18.90
${\mathrm{IR}-S}_{\mathrm{ju}}R_{a}^{t}Z$	$\pi_{\mathrm{ad}},\varphi_{\mathrm{ju}}^{12}(t),\varphi_{\mathrm{ad}}^{12}(t+\gamma_{\mathrm{ad}}),p(t+\mathrm{cov})$	Max air temperature	52	−268.09	19.12
${\mathrm{IR}-S}_{\mathrm{ju}}R_{a}^{t}C$	$\pi_{\mathrm{ad}},\varphi_{\mathrm{ju}}^{12}(t),\varphi_{\mathrm{ad}}^{12}(t+\gamma_{\mathrm{ad}}),p(c)$	‐	51	−269.73	20.39
${\mathrm{IR}-S}_{\mathrm{ju},\mathrm{ad}}R_{a}^{t}Z$	$\varphi_{\mathrm{ju}}^{12}(t),\varphi_{\mathrm{ad}}^{12}(t+\gamma_{\mathrm{ad}}),p(t+\mathrm{cov})$	Max air temperature	51	−270.71	22.36
${\mathrm{IR}-S}_{\mathrm{ju},\mathrm{ad}}R_{a}^{t}Z$	$\varphi_{\mathrm{ju}}^{12}(t),\varphi_{\mathrm{ad}}^{12}(t+\gamma_{\mathrm{ad}}),p(t+\mathrm{cov})$	Min air temperature	51	−271.21	23.36
	GRM model				
G–Z	p(t+cov)	Precipitation	4	−387.83	161.40
G–C	p(c)	‐	3	−390.05	163.84

All of the top 10 IRMER models ranked by AIC include fully time‐dependent transition probabilities $\varphi_{i}^{12}$ for juvenile individuals. As there are many boundary estimates of transition probabilities, as shown in Web Appendix E, we computed standard errors and confidence intervals empirically using non‐parametric bootstrap (500 resamples). The procedure for the non‐parametric bootstrap is described in Web Appendix D. The estimate of the number of individuals not captured is 57.74 (SE 146.02, 95% bootstrap CI 31.75, 473.33) for juveniles and 3.36 (SE 49.76, 95% bootstrap CI 0.02, 180.11) for adults. The poor precision of population sizes is likely due to the small sample sizes, low capture probability and low availability of individuals. The time‐varying capture probability is estimated to be 0.18 on average where the estimated intercept and slope of the logit are 1.98 (SE 0.60, 95% bootstrap CI −3.60, −1.46) and 1.61 (SE 0.37, 95% bootstrap CI 0.98, 2.32), respectively. The estimated additive effect of transition probabilities for adults is −0.97 (SE 1.54, 95% bootstrap CI −1.84, 2.75). The mean of $\hat{\varphi_{i}^{12}}$ are 0.70 and 0.57 for juveniles and adults, respectively, so the common lizards, we analyzed tend to stay in an unobservable state on average. The results for the transition probabilities are shown in Web Appendix E. Standard errors for the $\varphi_{i}^{12}$ are large for some primary sessions. The complexity of the model can be reduced by defining the fully time‐dependent $\varphi_{i}^{12}$ to follow parametric distributions such as a Beta distribution. However, no improvement in relative fit to the common lizard data is observed.

A visual assessment of observed and expected numbers provided no evidence of systematic lack of fit of the selected model (see Web Appendix E). Juveniles exhibit more powers of dispersal than adults as they can rapidly colonize new habitats which often become available adjacent to already occupied sites (Edgar et al., 2010). These characteristics are supported by the results from our top model, suggesting that the $\varphi_{i}^{12}$ of juveniles are higher than for adults. None of the available covariates collected during the study adequately accounted for the time‐dependent transition probabilities. However, the logistic regression of time‐varying capture probabilities in terms of precipitation is supported by our top ranked model.

We also considered the GRM model in Table 3, where G–C and G–Z represent the geometric removal model with constant and time‐varying capture probabilities in terms of covariates respectively, where the same capture probability at each sampling occasion for both populations is assumed. The estimate of the population size obtained by the G–Z model is 161.12 (SE 52.00, 95% bootstrap CI 51.91, 230.41) for juveniles and 34.90 (SE 18.41, 95% bootstrap CI 0.01, 71.65) for adults. The GRM models give larger estimates for the sizes of both populations.

## 6 Discussion

Removal models have considerable potential to inform the design and execution of removals of protected/invasive species, but need to take into account temporary emigration to reduce the risk of biased estimates of the number of animals not captured. We have extended the classic removal model to accommodate a robust design sampling strategy and multi‐event framework with one unobservable state. Our work is motivated by real data from translocation projects and it could also be adopted for removals of invasive species. Simulations and theoretical parameter redundancy assessment have demonstrated that the RMER models perform better than MER models. Our approaches yield unbiased estimates of the number of individuals in the populations residing in the sampling area when the sample size is large enough.

The adequate design of sampling protocols is fundamental at the data analysis stage. Mitigating a problem with study design is always highly recommended as the design of a study will govern how data can be analyzed. In this article, we have demonstrated that the use of the robust design for removal data can overcome issues of parameter redundancy and enable the estimation of transition probabilities between observable and unobservable states at a single study site. In addition, RMER models result in estimators of population size and capture probability which have better properties than MER under the standard sampling protocol. Therefore, we would like to raise the awareness of good study design for removal experiments as in our experience only a small number of removal studies have repeated samplings conducted within a day; however if sampling strategies were simply altered to allow for multiple secondary samples, uncertainty in estimates of detection and transition probabilities would reduce considerably.

The general RMER model with fully time‐dependent parameters is parameter redundant. Although the assumption of constant parameters across time is the most straightforward way of constraining models in order to enable estimation, using simulation we have demonstrated that the best performing models with least bias incorporate at least two constraints ’ constraint “$R^{t}$”, which denotes random emigration, and constraint “S,” which denotes that the initial state parameter π is constrained as the first element of the mean of the stationary distributions of the transition matrices across time.

Our proposed RMER model is general and can be extended to the IRMER modeling approach which permits the analysis of multiple data sources, exploiting the relationship between parameters expected between related populations. Furthermore, we have applied the IRMER model to two age groups (adults and juveniles) of common lizard data and the results align with our understanding of the natural history of this species.

We define the population size *N* as the total number of individuals that could possibly be captured across the study. However, if some individuals are unlikely to enter the study area and are unavailable for capture over the whole duration of the study, then they cannot be included in the estimate. Practical aspects of study design such as an efficient distribution of traps to make sure the arrangement of traps exposes as many individuals as possible, should be considered, to overcome this issue as much as is feasible.

As permanent departure from the population is possible during the study, it may be useful to model mortality and temporary emigration simultaneously. We have considered a constant survival probability as an extra parameter in our proposed model in Web Appendix F. We find that the ${R-\mathrm{SR}}_{t}$C model with a survival probability is full‐rank but parameters are not identifiable for most of the simulation scenarios due to near redundancy. Therefore, we assume that all individuals survive when analyzing the real data as translocation studies are usually conducted over a relatively short period of time (up to months). Improved estimates of survival probability can be obtained from removal data by collecting ancillary information during removal sampling, for example, concurrent capture–recapture sampling as suggested in Gould and Pollock (1997) or a few capture–recapture sampling occasions prior to removal sampling which is a design we are currently investigating.

Spatial information has been widely used in the capture recapture literature (Royle et al., 2013), however, there is no spatial information on sampling available for the real data. Translocation projects are generally poorly documented globally. In the UK, less than 10% of submitted reports contain detailed population monitoring data and one‐half of the cases on file lack any type of report (Germano et al., 2015). In order to optimize the success of translocation studies, we should not only design the study properly, but also record any informative component which may help evaluate the sampling methodologies.

## 7 Supplementary Materials

Web Appendices A (Parameter redundancy), B (Simulations results), C (Investigation of the relaxation of constraint “R”), D (Non‐parametric bootstrap for removal data), E (Results for Data Analysis) and F (Consider mortality in the ${R-\mathrm{SR}}^{t}$C model) referenced in Sections 3, 4, 2, 5, 5, and 6 respectively, together with selected computer R code implementing the proposed model and Maple code for detecting parameter redundancy are available with this article at the *Biometrics* website on Wiley Online Library.

## Supporting information

Supplementary Data Code S1.

Supplementary Data S1.
