# Supplementary material for: Removal models accounting for temporary emigration
Source: Biometrics. 2018 Sep 19;75(1):24–35. doi: 10.1111/biom.12961 (PMC7379670; doi:10.1111/biom.12961)
Supplement: Supplementary file 2 — Supplementary Data S1. [file BIOM-75-24-s002.pdf]

Web-based Supplementary Materials for Removal Models Accounting for Temporary Emigration

Zhou, M., McCrea, R. S., Matechou, E., Cole, D. J. and Griffiths., R. A.

June 15, 2018

Contents

1 Web Appendix A: Parameter redundancy **1**
1.1 Methods for detecting parameter redundancy results 2
1.1.1 NNC example 2
1.2 Generalization of parameter redundancy results 3
1.2.1 Extension theorem 3
1.2.2 Reparameterization theorem 4
1.3 Near parameter redundancy 5

2 Web Appendix B: Simulation results **8**
2.1 Results under *Setting 4.1* 8
2.1.1 Results under Scenarios 1-4 8
2.1.2 Results under Scenarios 5-8 11
2.1.3 Results for the GRM model 12
2.2 Results under *Setting 4.2* 14
2.2.1 Results under Scenarios 1-4 14
2.2.2 Results under Scenarios 5-8 17
2.2.3 Results for the GRM model 19
2.3 Results under *Setting 4.3* 21
2.3.1 Results under Scenarios 1-4 21
2.3.2 Results for the GRM model 25

3 Web Appendix C: Investigation of the relaxation of constraint "R^{t}" **26**

4 Web Appendix D: Non-parametric bootstrap for removal data **29**

5 Web Appendix E: Results for Data Analysis **31**

6 Web Appendix F: Consider mortality in the R-SR^{t}C model **35**

1 Web Appendix A: Parameter redundancy

A model is called parameter redundant when parameters in the model are confounded, so that the model could be reparameterized in terms of a small number of parameters (Catchpole and Morgan, 1997). In Section 3 of the main paper we discussed parameter redundancy of different removal models. Here in Section 1.1 we provide more detail on how to detect parameter redundancy for a fixed number of sampling occasions, giving an illustrative example in Section 1.1.1. Then in Section 1.2 we show how to generalize parameter redundancy results for any number of sampling occasions. Theoretically we can estimate all the parameters in a model that is not parameter redundant. However, good performance of the model is not guaranteed if the model is in same way close to a model that is parameter redundant; this is known as near redundancy. Near redundancy is discussed in Section 1.3.

1.1 Methods for detecting parameter redundancy results

We have briefly described how to determine whether or not the model is parameter redundant in Section 3 of the main paper. This involves forming a derivative matrix,

$$\mathbf{D} = \left[\frac{\partial \boldsymbol{\kappa}(\boldsymbol{\theta})}{\partial \boldsymbol{\theta}}\right]$$

where $\boldsymbol{\theta}$ is a vector of h parameters and where $\boldsymbol{\kappa}(\boldsymbol{\theta})$ is a vector of parameter combinations that uniquely represent the model. This is known as an exhaustive summary (Cole et al., 2010). For removal models a suitable exhaustive summary is a vector of probabilities of individuals being removed at the kth occasion, for $k = 1, \ldots, K$. This is similar to the exhaustive summary used in Cole et al. (2012).

The deficiency of the model, d, is calculated as $h - r$, where r is the rank of the derivative matrix. If $d > 0$, the model is parameter redundant. Otherwise, the model is termed full rank and is not parameter redundant. For a parameter redundant model, it is possible to show, if any, of the original parameters are estimable by solving $\boldsymbol{\alpha}^T\mathbf{D} = 0$, for a vector $\boldsymbol{\alpha}(\boldsymbol{\theta})$. There will be d solution to $\boldsymbol{\alpha}^T\mathbf{D} = 0$. If we denote entries of the d solutions as $\alpha_{i,j}$, for $i = 1, \ldots, h$ and $j = 1, \ldots, d$, then any zero $\alpha_{i,j}$ for all j corresponds to an estimable parameter. To find other estimable parameter combinations, we solve the system of linear first-order partial differential equations, $\sum_{i=1}^{v} \alpha_{i,j} \partial f / \partial \theta_i = 0$, where $j = 1, \ldots, d$ (Catchpole et al., 1998; Cole et al., 2010).

We demonstrate how this method works using an illustrative example in Section 1.1.1 below.

Note that all of the parameter redundancy results in Tables 1 and 2 in the paper are based on the assumption that at least one animal is removed at every sampling occasion. This ensures that all of the exhaustive summary terms are present as we are interested in detecting parameter redundancy in the model itself instead of parameter redundancy due to the lack of data.

1.1.1 NNC example

The NNC model in Table 1 in the main paper considers a multievent framework allowing for temporary emigration but does not have a robust design structure. It contains a constant detection probability, constant transition probabilities and an initial state parameter. Therefore, the parameters in this case are $\boldsymbol{\theta} = [p \quad \phi^{12} \quad \phi^{21} \quad \pi]$, where p is the detection probability, ϕ^{12} and ϕ^{21} represents transition probabilities between state 1 and state 2, and π is the probability of being in the state 1 initially. Consider the model with 8 occasions of removal, and we define the exhaustive summary as a vector of probabilities of an individual being captured at the the kth occasion as the specification determines the model. We only show first three terms in the probability matrix below as the probabilities of removal dramatically get more complicated as the number of sampling times increase. All the `Maple` files are available in the supplementary material.

$$\boldsymbol{\kappa}(\boldsymbol{\theta}) = \left[L_{k,1}\right] =$$

$$\begin{bmatrix} \pi p \\ \{\pi(1-p)(1-\phi^{12}) + (1-\pi)\phi^{21}\}p \\ \left[\{\pi(1-p)(1-\phi^{12}) + (1-\pi)\phi^{12}\}(1-p)(1-\phi^{12}) + \{\pi(1-p)\phi^{12} + (1-\pi)(1-\phi^{21}\}\phi^{21})\right]p \\ \vdots \end{bmatrix}$$

where $L_{k,1}$ is the probability of animals being captured at the kth sampling occasion. We remove the more complicated probability that an animal is never captured to make the exhaustive summary simpler, as we assume we observe at least one individual for all possible other capture histories and all the probabilities sum up to one (Catchpole and Morgan, 1997).

The derivative matrix is given by,

$$\mathbf{D} = \left[\frac{\partial \boldsymbol{\kappa}(\boldsymbol{\theta})}{\partial \boldsymbol{\theta}}\right] = \begin{bmatrix} \pi & -\pi(1-\phi^{12})p + \pi(1-p)(1-\phi^{12}) + (1-\pi)\phi^{21} & \cdots \\ 0 & -\pi(1-p)p & \cdots \\ 0 & (1-\pi)p & \cdots \\ p & \{(1-p)(1-\phi^{12}) - \phi^{21}\}p & \cdots \end{bmatrix}.$$

and is found to have symbolic rank 3. However, there are 4 parameters, therefore the model is

parameter redundant with deficiency 1. In order to find if any of the original parameter can be estimated, we solve $\boldsymbol{\alpha}^T\mathbf{D} = 0$, which gives,

$$\boldsymbol{\alpha} = \begin{bmatrix} -\dfrac{p}{\pi} & \dfrac{p\phi^{12} - p + \phi^{21}}{\pi(p-1)} & \dfrac{\phi^{21}}{\pi} & 1 \end{bmatrix}.$$

As there are no zeros in $\boldsymbol{\alpha}$, none of the original parameters are individually estimable. To find the estimable parameter combinations, we solve the partial differential equation,

$$-\frac{\partial f}{\partial p}\frac{p}{\pi} + \frac{\partial f}{\partial \phi^{12}}\frac{p\phi^{12} - p + \phi^{21}}{\pi(p-1)} + \frac{\partial f}{\partial \phi^{21}}\frac{\phi^{21}}{\pi} + \frac{\partial f}{\partial \pi} = 0.$$

The solution of which shows we can estimate $p\phi^{21}$, πp, and $p(\phi^{12} - 1) - \phi^{12} - \phi^{21}$.

1.2 Generalization of parameter redundancy results

In Section 1.1.1, we demonstrate how to determine the results of parameter redundancy for a fixed number of K sampling occasions with two secondary samples within each primary period. It is possible to generalize the results of parameter redundancy to any number of samples as described in Catchpole and Morgan (1997) and Cole et al. (2010). In this section, we show how to obtain general parameter redundancy results for our removal models with two secondary occasions within each primary period. Note that we could theoretically generalize the results to any number of secondary occasions. Because in this paper the data collected has two secondary occasions within each primary period, so we demonstrate the generalization of parameter redundancy results for data with two secondary occasions.

1.2.1 Extension theorem

Parameter redundancy results can be generalised to any dimension of model using the extension Theorem. The extension Theorem was first proposed by Catchpole and Morgan (1997)and extended to any exhaustive summary in (Cole et al., 2010).

Suppose we have a full rank model for a fixed number of total sampling occasions, in which its exhaustive summary is denoted by $\boldsymbol{\kappa}_1(\boldsymbol{\theta}_1)$ with parameters $\boldsymbol{\theta}_1$ and its derivative matrix is $\mathbf{D}_{1,1} = [\partial\boldsymbol{\kappa}_1(\boldsymbol{\theta}_1)/\partial\boldsymbol{\theta}_1]$. The model is then extended, by adding an extra sampling occasion, to give exhaustive summary $\boldsymbol{\kappa} = [\boldsymbol{\kappa}_1, \boldsymbol{\kappa}_2]$. We denote the extra parameters (if any) by $\boldsymbol{\theta}_2$ and let $\boldsymbol{\theta} = [\boldsymbol{\theta}_1, \boldsymbol{\theta}_2]$. The extra exhaustive summary terms are $\boldsymbol{\kappa}_2(\boldsymbol{\theta})$. Furthermore, the new derivative matrix of the extended model is formed by,

$$\mathbf{D} = \begin{bmatrix} \mathbf{D}_{1,1}(\boldsymbol{\theta}_1) & \mathbf{D}_{2,1}(\boldsymbol{\theta}_1) \\ \mathbf{D}_{1,2}(\boldsymbol{\theta}_2) & \mathbf{D}_{2,2}(\boldsymbol{\theta}_2) \end{bmatrix} = \begin{bmatrix} \mathbf{D}_{1,1}(\boldsymbol{\theta}_1) & \mathbf{D}_{2,1}(\boldsymbol{\theta}_1) \\ 0 & \mathbf{D}_{2,2}(\boldsymbol{\theta}_2) \end{bmatrix},$$

where $\mathbf{D}_{1,2}(\boldsymbol{\theta}_2) = [\partial\boldsymbol{\kappa}_1/\partial\boldsymbol{\theta}_2] = 0$, $\mathbf{D}_{2,1}(\boldsymbol{\theta}_1) = [\partial\boldsymbol{\kappa}_2/\partial\boldsymbol{\theta}_1]$ and $\mathbf{D}_{2,2}(\boldsymbol{\theta}_2) = [\partial\boldsymbol{\kappa}_2/\partial\boldsymbol{\theta}_2]$.

Catchpole and Morgan (1997) proved that if both $\mathbf{D}_{1,1}(\boldsymbol{\theta}_1)$ and $\mathbf{D}_{2,2}(\boldsymbol{\theta}_2)$ are full rank, then the extended model is also full rank. If there is no extra parameter of the extended model, then the new derivative matrix can be further simplified to,

$$\mathbf{D} = \begin{bmatrix} \mathbf{D}_{1,1}(\boldsymbol{\theta}_1) & \mathbf{D}_{2,1}(\boldsymbol{\theta}_1) \\ 0 & \mathbf{D}_{2,2}(\boldsymbol{\theta}_2) \end{bmatrix} = \begin{bmatrix} \mathbf{D}_{1,1}(\boldsymbol{\theta}_1) & \mathbf{D}_{2,1}(\boldsymbol{\theta}_1) \end{bmatrix},$$

which is always full rank if $\mathbf{D}_{1,1}(\boldsymbol{\theta}_1)$ is full rank.

Furthermore, if there is only one extra parameter in the extended model, then $\mathbf{D}_{2,2}(\boldsymbol{\theta}_2)$ is always full rank because it always has rank of one as a row vector. Therefore the extended model will be full rank if $\mathbf{D}_{1,1}(\boldsymbol{\theta}_1)$ is full rank and there is only one new parameter in $\boldsymbol{\theta}_2$. Therefore, the model is always full rank by induction. The use of extension theorem is demonstrated in the `Maple` file for the R-SR^{t}C model in Table 2 in the paper.

Web Table 1: Parameter redundancy results for parameter redundant RMER models in the Table 2 in the paper, where ϕ_i^{12} and ϕ_i^{21} are time-varying over time. The parameter redundancy results hold for an even number of total sampling occasions K, where there are two secondary samples for each primary period. d is the deficiency of the model, calculated as the number of parameters minus the rank of the derivative matrix. EP represents the estimable combinations of parameters if the model is parameter redundant. "t" denotes time-dependence, "c" denotes a constant parameter.

Model code	Model	d	EP
R-NN^tC	$\pi, \phi^{12}(t), \phi^{21}(t), p(c)$	$K/2-1$	p, π,<br>$f(p, \pi, \phi_1^{12}, \phi_1^{21})$,<br>$\cdots$,<br>$f(p, \pi, \phi_1^{12}, \phi_1^{21}, \cdots, \phi_{K/2-1}^{12}, \phi_{K/2-1}^{21})$
R-NN^tZ	$\pi, \phi^{12}(t), \phi^{21}(t), p(t+\text{cov})$	$K/2-1$	α, β, π,<br>$f(\alpha, \beta, \pi, \phi_1^{12}, \phi_1^{21})$,<br>$\cdots$,<br>$f(\alpha, \beta, \pi, \phi_1^{12}, \phi_1^{21}, \cdots, \phi_{K/2-1}^{12}, \phi_{K/2-1}^{21})$
R-SN^tC	$\phi^{12}(t), \phi^{21}(t), p(c)$	$K/2-2$	p,<br>$f(\phi_1^{12}, \phi_1^{21}, \cdots, \phi_{K/2-1}^{12}, \phi_{K/2-1}^{21})$,<br>$f(p, \phi_1^{12}, \phi_1^{21})$,<br>$\cdots$,<br>$f(p, \phi_1^{12}, \phi_1^{21}, \cdots, \phi_{K/2-1}^{12}, \phi_{K/2-1}^{21})$
R-N2^tC	$\pi, \phi^{12}(t), \phi^{21}(t), p(c)$	$K/2-3$	p, π, $\phi_{K/2-2}^{12}$, $\phi_{K/2-2}^{21}$,<br>$f(p, \pi, \phi_1^{12}, \phi_1^{21})$,<br>$\cdots$,<br>$f(p, \pi, \phi_1^{12}, \phi_1^{21}, \cdots, \phi_{K/2-2}^{12}, \phi_{K/2-2}^{21})$
R-SN^tZ	$\phi^{12}(t), \phi^{21}(t), p(t+\text{cov})$	$K/2-2$	α, β,<br>$f(\phi_1^{12}, \phi_1^{21}, \cdots, \phi_{K/2-1}^{12}, \phi_{K/2-1}^{21})$,<br>$f(\alpha, \beta, \phi_1^{12}, \phi_1^{21})$,<br>$\cdots$,<br>$f(\alpha, \beta, \phi_1^{12}, \phi_1^{21}, \cdots, \phi_{K/2-1}^{12}, \phi_{K/2-1}^{21})$
R-N2^tZ	$\pi, \phi^{12}(t), \phi^{21}(t), p(t+\text{cov})$	$K/2-3$	α, β, π, $\phi_{K/2-2}^{12}$, $\phi_{K/2-2}^{21}$),<br>$f(\alpha, \beta, \pi, \phi_1^{12}, \phi_1^{21}$,<br>$\cdots$,<br>$f(\alpha, \beta, \pi, \phi_1^{12}, \phi_1^{21}, \cdots, \phi_{K/2-2}^{12}, \phi_{K/2-2}^{21})$

1.2.2 Reparameterization theorem

If the original model is not full rank, then the extension theorem cannot be used for that model. Instead, we need to find a reparameterization of the original model which is full rank to begin with, then we can apply the extension theorem to the reparameterized model to determine its the general parameter redundancy result (Cole et al., 2010). We reparameterize our models in terms of the estimable parameter combinations. The deficiency and estimable parameter combinations are derived explicitly in Web Table 1 which provides additional detail on the estimable parameter combination for the parameter redundant models in the Table 2 in the main paper. We demonstrate how to use the reparameterization theorem for the model R-NN^{t}C in Table 2 in the paper in the `Maple` file.

1.3 Near parameter redundancy

We have described near parameter redundancy for a full rank model in Section 3 of the paper. Following the method of detecting near redundancy in Catchpole et al. (2001), we calculated the expected information matrix in `Maple` and obtained the smallest standardized eigenvalues for the RMER models with constant capture probability in the paper, the standardized eigenvalues are computed as the absolute value of the ratio of the smallest eigenvalue and the largest eigenvalue. A very small eigenvalue in a full rank model indicates a problem with near redundancy. The results are showed in Web Tables 2-5, where we described Scenarios 1-2 and Scenarios 3-8 in the main paper and in Section 2 in the Web Appendix B respectively.

R-NNC has extremely small standardized eigenvalues across all scenarios in Web Table 2, which results in biased parameter estimates in the simulation studies given in Figures 1 in the paper, Web Figures 1. In Web Table 3 we show that the standardized smallest eigenvalues for the R-SEC model are larger than those in the R-NEC model. Also, We observe similar results in the simulation study in Section 2.1.2, where the performance of R-SEC is better than R-NEC under each Scenario shown in Web Figure 2.

In Web Table 4, the R-SR$_2^t$C and R-SR^{t}C have largest two standardized eigenvalues under each scenario, which suggests these two models behave better than the rest of the models. It matches what we found in Web Figures 5 and 6 in Section 2.2.1. Also, relatively smaller standardized eigenvalues for R-NR^{t}C model in Web Table 4 explain its poor behaviours in Web Figures 5-6. Although we observe relatively large standardized eigenvalues for models in Web Table 5 especially under Scenarios 6 and 8, none of these models yield unbiased results for time-dependant transition probabilities in Web Figures 8.

We also notice there is no clear cut-off point between large and small standardized eigenvalues across all Scenarios in our cases. So, we suggest that any interpretations of standardized eigenvalues have to be made cautiously.

Web Table 2: Results of standardized smallest eigenvalues (S.Eig) for R-NNC, R-SNC, R-NRC and R-SRC models in Table 1 in the paper under Scenarios 1-4. All models are diagnosed with $K = 8$ sampling occasions, where there are $T = 4$ primary periods and $k_1 = k_2 = k_3 = k_4 = 2$ secondary samples for each primary period.

Scenario	Model	S.Eig
Scenario 1	R-NNC	1.03×10^{-11}
	R-SNC	3.06×10^{-3}
	R-NRC	1.67×10^{-2}
	R-SRC	1.58×10^{-2}
Scenario 2	R-NNC	6.25×10^{-12}
	R-SNC	9.86×10^{-3}
	R-NRC	1.39×10^{-2}
	R-SRC	2.24×10^{-2}
Scenario 3	R-NNC	1.34×10^{-12}
	R-SNC	1.55×10^{-2}
	R-NRC	8.61×10^{-2}
	R-SRC	7.85×10^{-2}
Scenario 4	R-NNC	1.34×10^{-12}
	R-SNC	9.96×10^{-2}
	R-NRC	1.29×10^{-1}
	R-SRC	2.19×10^{-1}

Web Table 3: Results of standardized smallest eigenvalues (S.Eig) for R-NEC and R-SEC models under Scenarios 5-8. All models are diagnosed with $K = 8$ sampling occasions, where there are $T = 4$ primary periods and $k_1 = k_2 = k_3 = k_4 = 2$ secondary samples for each primary period.

Scenario	RMER Model	S.Eig
Scenario 5	R-NEC	7.47×10^{-3}
	R-SEC	2.23×10^{-2}
Scenario 6	R-NEC	9.48×10^{-3}
	R-SEC	6.27×10^{-2}
Scenario 7	R-NEC	9.10×10^{-2}
	R-SEC	1.72×10^{-2}
Scenario 8	R-NEC	1.35×10^{-1}
	R-SEC	4.84×10^{-1}

Web Table 4: Results of standardized smallest eigenvalues (S.Eig) for R-NR^{t}C, R-SR^{t}C, R-NR$_2^t$C and R-SR$_2^t$C models under Scenarios 1-4. All models are diagnosed with $K = 8$ sampling occasions, where there are $T = 4$ primary periods and $k_1 = k_2 = k_3 = k_4 = 2$ secondary samples for each primary period.

Scenario	Model	S.Eig
Scenario 1	R-NR^{t}C	2.02×10^{-2}
	R-SR^{t}C	2.12×10^{-2}
	R-NR$_2^t$C	1.98×10^{-2}
	R-SR$_2^t$C	2.61×10^{-1}
Scenario 2	R-NR^{t}C	2.82×10^{-3}
	R-SR^{t}C	5.06×10^{-3}
	R-NR$_2^t$C	4.42×10^{-3}
	R-SR$_2^t$C	4.30×10^{-2}
Scenario 3	R-NR^{t}C	1.12×10^{-1}
	R-SR^{t}C	1.42×10^{-1}
	R-NR$_2^t$C	1.04×10^{-1}
	R-SR$_2^t$C	2.18×10^{-1}
Scenario 4	R-NR^{t}C	3.13×10^{-4}
	R-SR^{t}C	4.52×10^{-2}
	R-NR$_2^t$C	1.96×10^{-2}
	R-SR$_2^t$C	7.73×10^{-2}

Web Table 5: Results of standardised smallest eigenvalues (S.Eig) for R-NEC, R-SEC and R-NE_2C models under Scenarios 5-8. All models are diagnosed with $K = 8$ sampling occasions, where there are $T = 4$ primary periods and $k_1 = k_2 = k_3 = k_4 = 2$ secondary samples for each primary period.

Scenario	Model	S.Eig
Scenario 5	R-NE^{t}C	1.37×10^{-4}
	R-SEC	1.02×10^{-3}
	R-SE$_2^t$C	1.15×10^{-3}
Scenario 6	R-NE^{t}C	2.43×10^{-2}
	R-SE^{t}C	3.92×10^{-2}
	R-SE$_2^t$C	2.58×10^{-2}
Scenario 7	R-NE^{t}C	1.09×10^{-3}
	R-SE^{t}C	4.46×10^{-3}
	R-SE$_2^t$C	3.70×10^{-3}
Scenario 8	R-NE^{t}C	1.84×10^{-1}
	R-SE^{t}C	2.09×10^{-1}
	R-SE$_2^t$C	1.67×10^{-1}

2 Web Appendix B: Simulation results

In this section, we provide more simulation results for our proposed models. Models with constraint "R" or "R^t" are investigated with low capture probability under Scenarios 1-2 as described in the paper, while simulations are conducted for high capture probability under Scenarios 3-4. In addition, simulations are carried out for constraint "E" or "E^t" under Scenarios 5-8 as shown below. The true values of capture probabilities p and transition probabilities ϕ_i^{12} for simulating the data under Scenarios 5-8 for the constraint "E" or "E^t" are same with those under Scenarios 1-4 described in the paper. Web Table 6 summarizes the true values of parameters we used for simulations for all scenarios we considered.

- Scenarios related to constraint "R" or "R^t':
    - Scenario 1: low capture probability and individuals tend to stay offsite ($\phi^{12} > 0.5$),
    - Scenario 2: low capture probability and individuals tend to stay onsite ($\phi^{12} < 0.5$),
    - Scenario 3: high capture probability and individuals tend to stay offsite ($\phi^{12} > 0.5$),
    - Scenario 4: high capture probability and individuals tend to stay onsite ($\phi^{12} > 0.5$).
- Scenarios related to constraint "E" or "E^t':
    - Scenario 5: low capture probability and high mobility ($\phi^{12} = \phi^{21} > 0.5$),
    - Scenario 6: low capture probability and low mobility ($\phi^{12} = \phi^{21} < 0.5$),
    - Scenario 7: high capture probability and high mobility ($\phi^{12} = \phi^{21} > 0.5$),
    - Scenario 8: high capture probability and low mobility ($\phi^{12} = \phi^{21} < 0.5$).

In addition to investigating the performance of the MER, RMER and IRMER models using simulation, we also run simulations for geometric removal models under each simulation settings. In Sections 2.1.3, 2.2.3 and 2.3.2 we show the results obtained from simulations for geometric removal models using data simulated with robust design under simulation *Setting 4.1 Setting 4.2* and *Setting 4.3* respectively. We observe extremely large estimates of population sizes and very tiny estimates of constant capture probabilities compare to the true values of parameters across all Scenarios. Therefore, we show that the geometric removal model overestimates the population size and underestimates the constant capture probability if temporary emigration and the robust design are ignored.

2.1 Results under *Setting 4.1*

We have discussed the simulation results in Figure 1-5 together with the results of near parameter redundant in Section 1.3. The best performing model is the R-SRC model.

2.1.1 Results under Scenarios 1-4

We have shown the results for the estimates of population size N, capture probability p and transition probability ϕ^{12} under Scenarios 1 and 2 in Figure 1 in the main paper. The results for the same models under Scenarios 3 and 4 are shown in Web Figure 1. Under Scenario 4 we nearly captured all of the individuals in the population so the estimates of population sizes are on the boundary of parameter spaces as shown in Web Figure 1(A). R-NNC model yields unbiased estimates of capture probability in Web Figure 1(B) while biased estimates of ϕ^{12} are observed in Web Figure 1(C) due to near parameter redundancy as discussed in Section 1.3.

Web Table 6: True values of parameters used for different simulation setting. Scenarios 1-4 are set for constraint "R". Scenarios 5-8 are set for constraint "E".

Setting	Model	K	Scenario	p	ϕ^{12}	λ_2	N	M
4.1	MER/RMER	10/20	1 (5)	0.3	0.8	-	500	-
			2 (6)	0.3	0.4	-	500	-
			3 (7)	0.6	0.8	-	500	-
			4 (8)	0.6	0.4	-	500	-
4.2	RMER	10	1 (5)	0.3	(0.7, 0.2, 0.7, 0.7)	-	500	-
			2 (6)	0.3	(0.3, 0.8, 0.3, 0.3)	-	500	-
			3 (7)	0.55	(0.7, 0.2, 0.7, 0.7)	-	500	-
			4 (8)	0.55	(0.3, 0.8, 0.3, 0.3)	-	500	-
		20	1 (5)	0.3	(0.8, 0.7, 0.8, 0.3, 0.6, 0.7, 0.8, 0.6, 0.6)	-	500	-
			2 (6)	0.3	(0.4, 0.4, 0.8, 0.4, 0.4, 0.8, 0.4, 0.4, 0.4)	-	500	-
			3 (7)	0.55	(0.8, 0.7, 0.8, 0.3, 0.6, 0.7, 0.8, 0.6, 0.6)	-	500	-
			4 (8)	0.55	(0.4, 0.4, 0.8, 0.4, 0.4, 0.8, 0.4, 0.4, 0.4)	-	500	-
4.3	IRMER	10	1 (5)	0.3	(0.7, 0.2, 0.7, 0.7)	-0.5	500	-
			2 (6)	0.3	(0.3, 0.8, 0.3, 0.3)	-0.5	500	-
			3 (7)	0.55	(0.7, 0.2, 0.7, 0.7)	-0.5	500	-
			4 (8)	0.55	(0.3, 0.8, 0.3, 0.3)	-0.5		
		20	1 (5)	0.3	(0.8, 0.7, 0.8, 0.3, 0.6, 0.7, 0.8, 0.6, 0.6)	-0.5	300	200
			2 (6)	0.3	(0.4, 0.4, 0.8, 0.4, 0.4, 0.8, 0.4, 0.4, 0.4)	-0.5	300	200
			3 (7)	0.55	(0.8, 0.7, 0.8, 0.3, 0.6, 0.7, 0.8, 0.6, 0.6)	-0.5	300	200
			4 (8)	0.55	(0.4, 0.4, 0.8, 0.4, 0.4, 0.8, 0.4, 0.4, 0.4)	-0.5	300	200

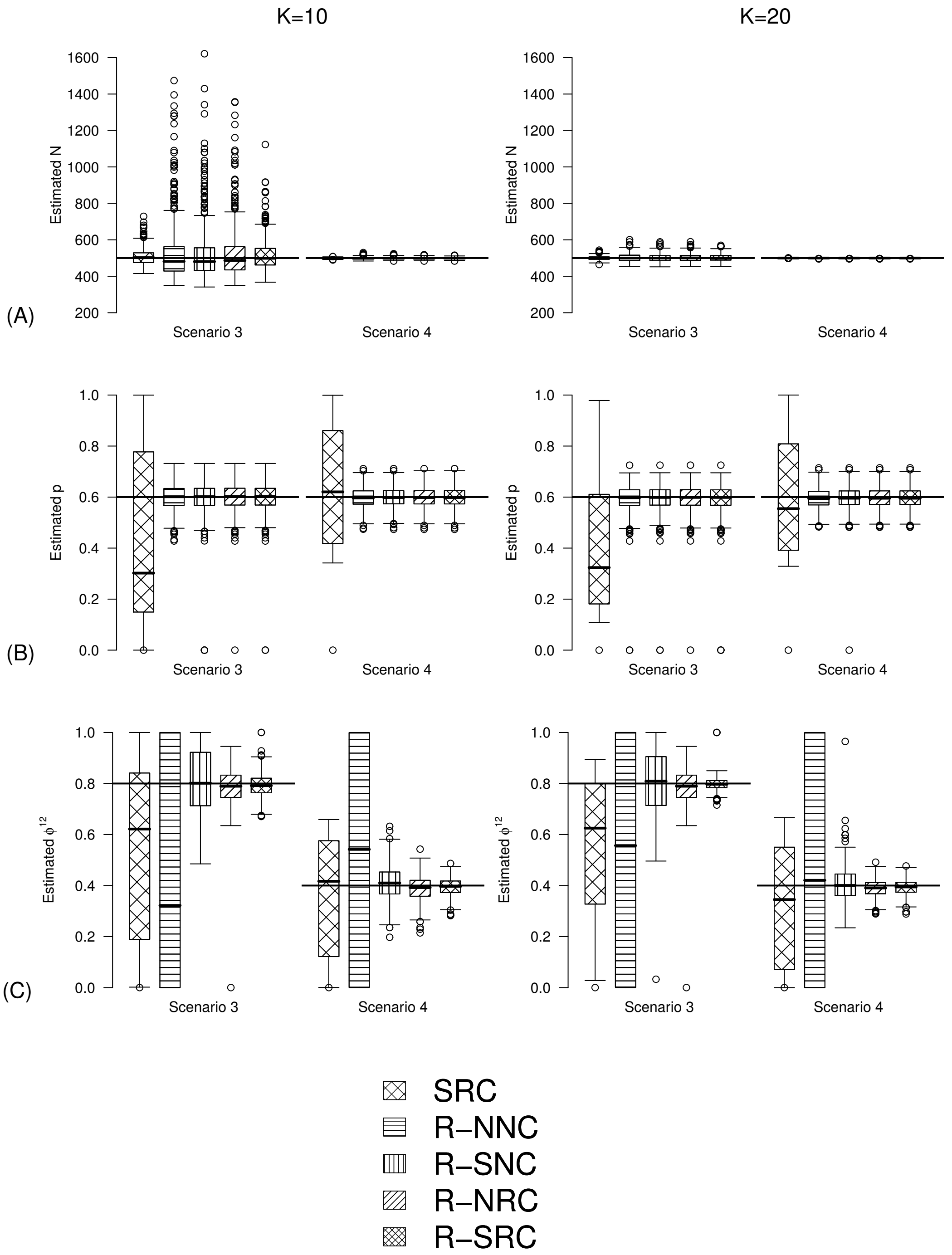

Web Figure 1: Results of estimated population size N (A), capture probability p (B) and transition probability ϕ^{12} (C) for simulations with $K = 10$ and $K = 20$ sampling occasions displayed on the first and second columns respectively. The black horizontal lines are the true values used for simulating the data under different scenarios. These models are presented in the Table 1 in the paper.

2.1.2 Results under Scenarios 5-8

In this Section, we show simulation results for models with constraint "E" in Table 1 in the main paper under Scenarios 5-8. When the population has high mobility under Scenarios 5 and 7, individuals are exposed to the study area more frequently, so nearly all of the individuals are removed from the population by the end of the study. Therefore, we observe boundary estimates of population sizes as shown in Web Figure 2(A). In Web Figure 2(B) the results of estimated capture probabilities are unbiased apart from those obtained from the NEC model. In Web Figure 2(C), unbiased estimates of ϕ^{12} are only obtained from the R-SEC model when we have $K = 20$ sampling occasions. However, the results in Section 2.2.2 suggest that biased estimated transition probabilities are obtained from the models incorporated with the constraint "E^t" when time-dependent transition probabilities are considered due to near parameter redundancy.

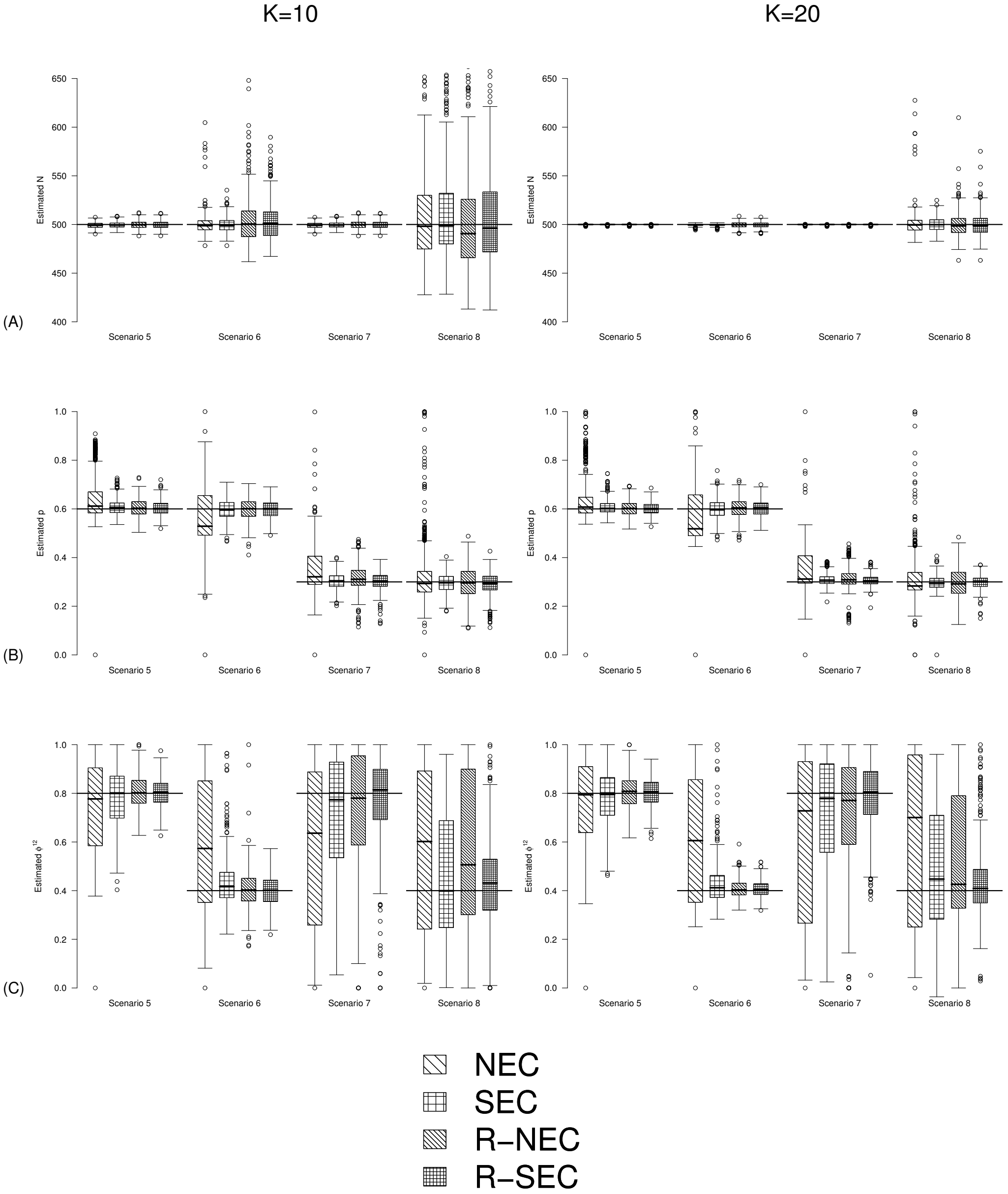

Web Figure 2: Results of estimated population size N (A) and capture probability p (B) and transition probability ϕ_{12} (C) for simulations with $K = 10$ and $K = 20$ sampling occasions displayed on the first and second columns respectively. These models are the corresponding models in Table 1 in the paper. The black horizontal lines are the true values for simulating the data under different scenarios.

2.1.3 Results for the GRM model

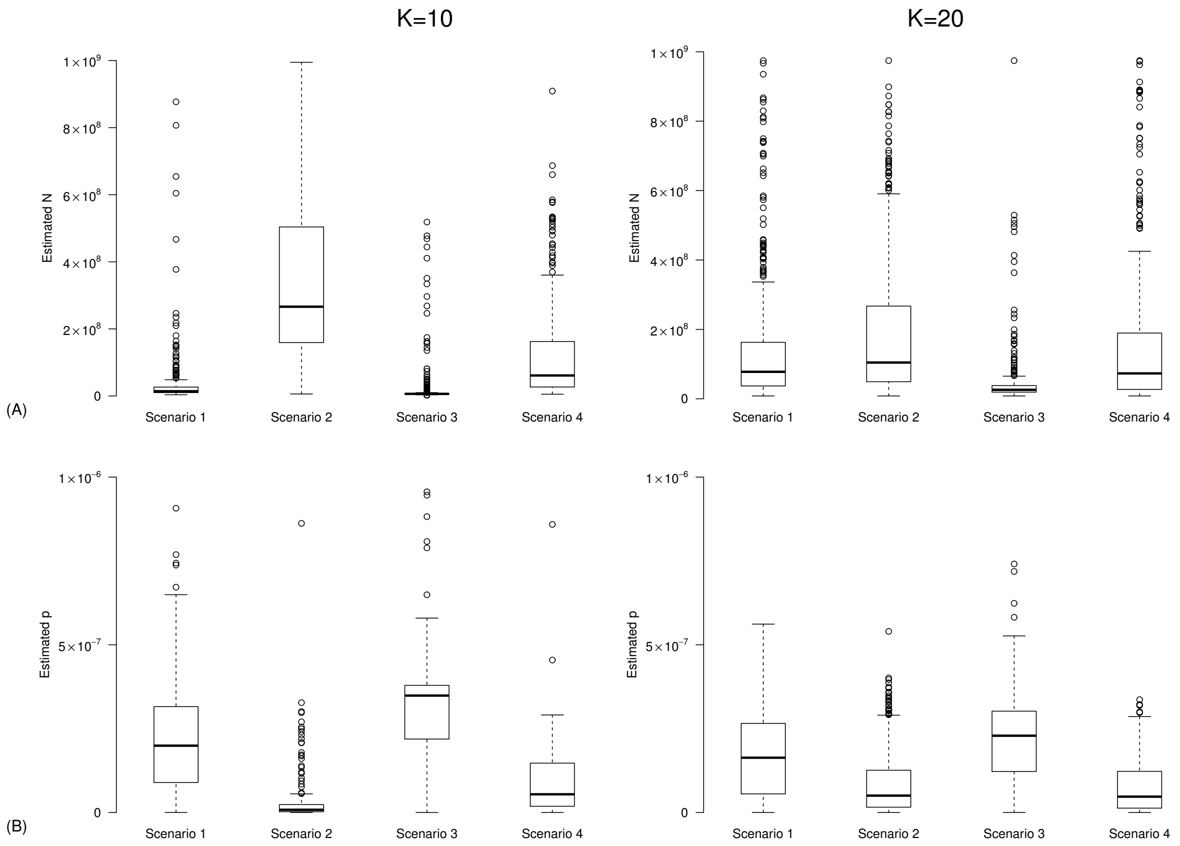

Web Figure 3: Results of estimated population sizes (A) and estimated capture probability p (B) for simulations with $K = 10$ and $K = 20$ sampling occasions displayed on the left and right column respectively. We fit the GRM models to the data simulated in Scenarios 1, 2, 3 and 4 under *Setting 4.1*. The true values for the capture probability is 0.3 under Scenarios 1 and 2, and 0.6 under Scenarios 3 and 4. In addition, the true value of the population size for simulating the data is $N = 500$.

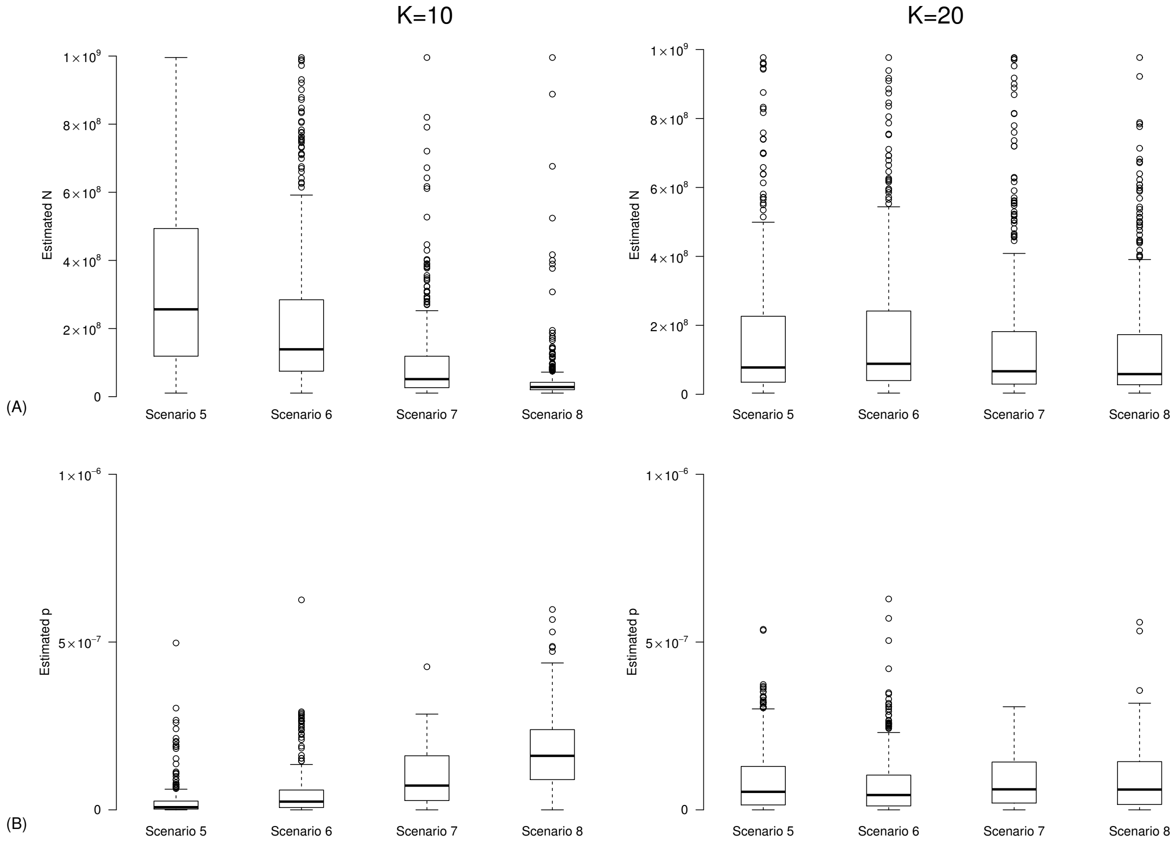

Web Figure 4: Results of estimated population sizes (A) and estimated capture probability p (B) for simulations with $K = 10$ and $K = 20$ sampling occasions displayed on the left and right column respectively. We fit the GRM models to the data simulated in Scenarios 5, 6, 7 and 8 under *Setting 4.1*. The true values for the capture probability is 0.3 under Scenarios 5 and 6, and 0.55 under Scenarios 7 and 8. In addition, the true value of the population size for simulating the data is $N = 500$.

2.2 Results under *Setting 4.2*

We described the simulation *Setting 4.2* in the main paper. We have discussed the simulation results in Figure 5-8 together with the results of near parameter redundancy in Section 3. The best performing model for time-dependent transition probabilities are the models with at least the constraint “S” and “R^t”, i.e. R-SR^tC and R-SR_2^tC models. None of the rest of model yield unbiased estimates for all of the parameters due to near parameter redundancy as discussed in Section 1.3.

2.2.1 Results under Scenarios 1-4

The results for the estimates of population size N, capture probability p and transition probability ϕ^{12} under Scenarios 1 and 2 are illustrated in Figure 2 in the main paper. The results for the estimated population sizes N and capture probabilities p for the same models under Scenarios 3 and 4 are shown in Web Figure 5. In Web Figure 6 we show the estimates of time-varying transition probabilities for Scenarios 1-4.

Web Figure 5 illustrates that unbiased estimates of population sizes are obtained from both the R-SR^tC and R-SR_2^tC models when there are 20 sampling occasions. However, the estimated population sizes are slightly biased low for R-SR^tC for 10 sampling occasions. In addition, R-NR^tC underestimates the population sizes due to near parameter redundancy as shown in Section 1.3. In Web Figure 6 we observe that the near parameter redundant R-NR^tC model underestimates most of the transition probabilities. Furthermore, the estimated transition probabilities ϕ_{12} between the last two primary periods obtained from the R-NR^tC model are on the boundary across all Scenarios.

Therefore we conclude that when modelling time-dependant transition probabilities, we need to consider at least the combination of the constraint “S” and “R^t”, otherwise the model is either parameter redundant or near parameter redundant.

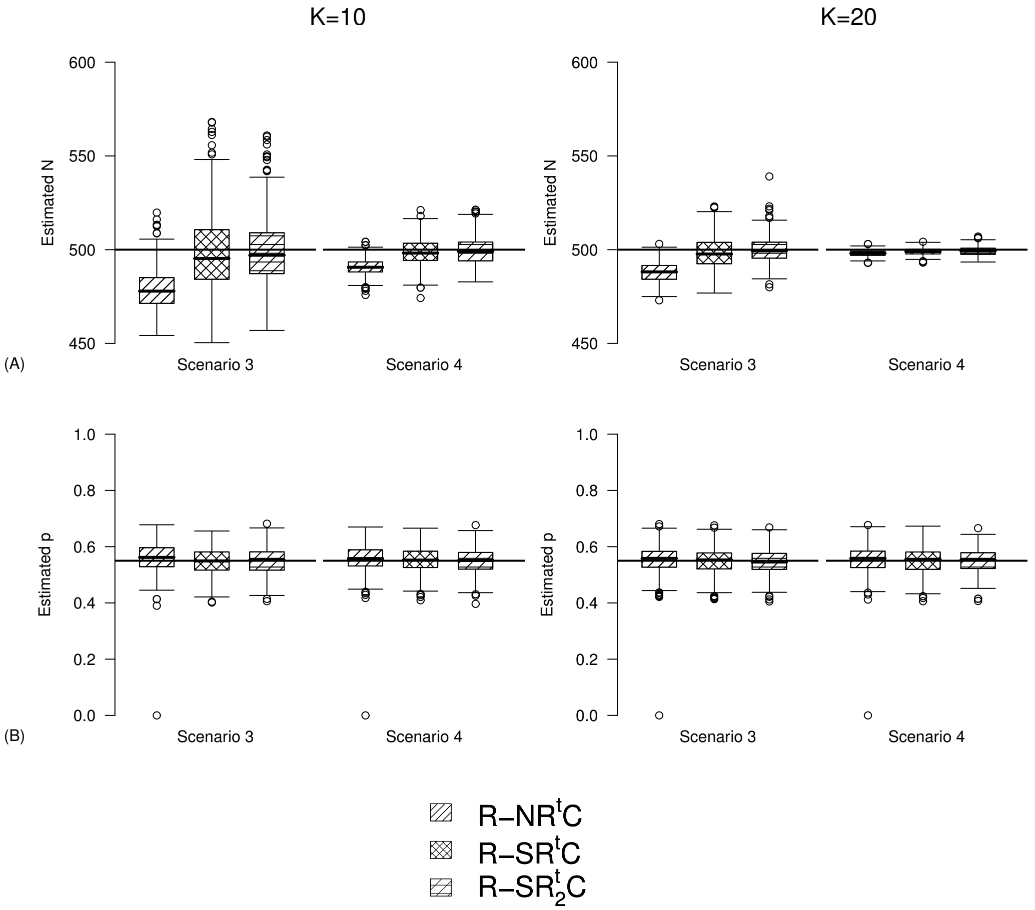

Web Figure 5: Results of estimated population sizs N (A) and capture probability p (B) for simulations with $K = 10$ and $K = 20$ sampling occasions displayed on the first and second columns respectively. These are the corresponding models in Table 2 in the paper. The black horizontal lines are the true values used for simulating the data under Scenarios 3 and 4.

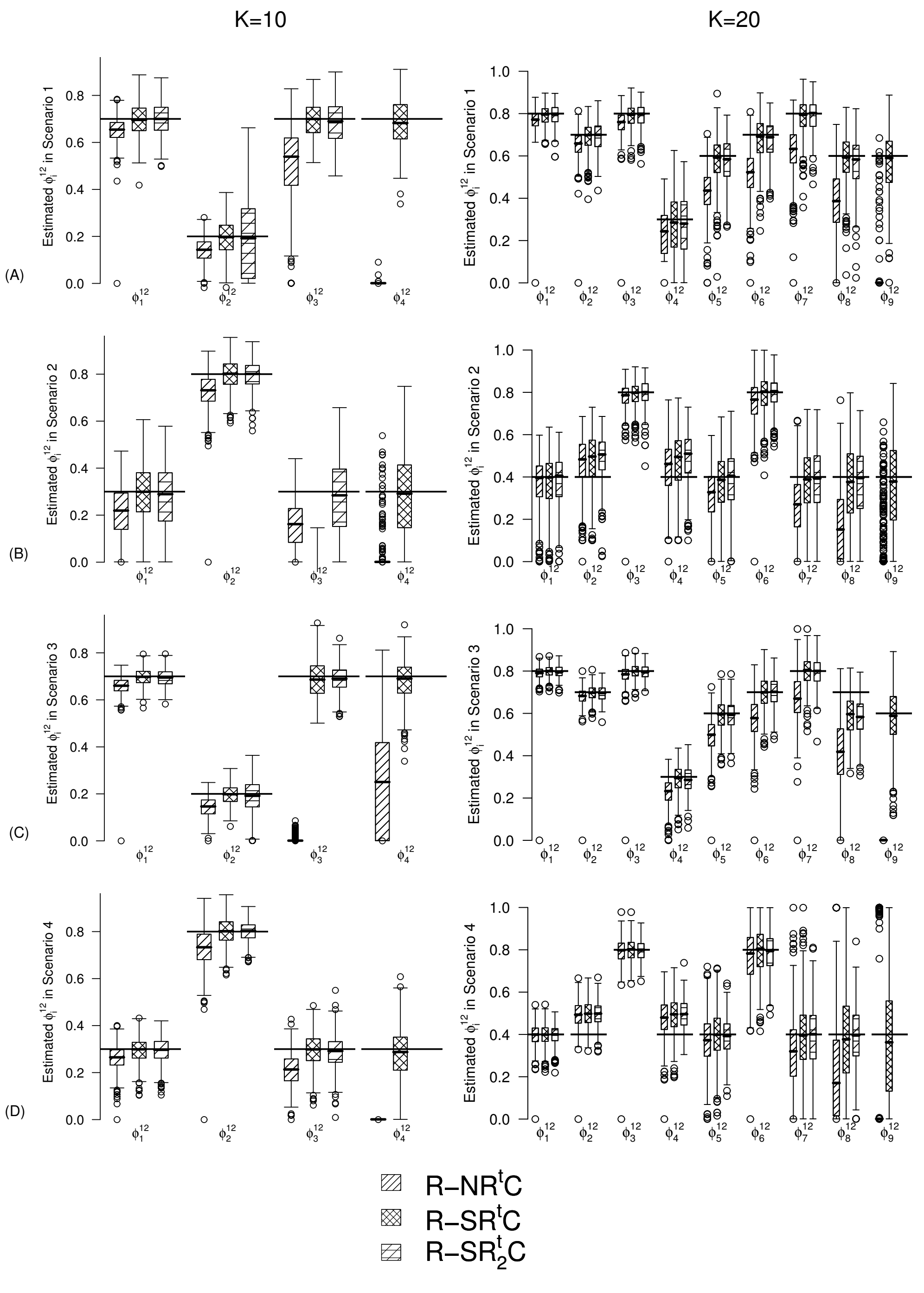

Web Figure 6: Estimated time-dependent transition probability ϕ_i^{12} for simulations with $K = 10$ and $K = 20$ sampling occasions. The black horizontal lines are the true values used for simulating the data under different scenarios. (A) Results of estimated ϕ_i^{12} under Scenario 1. (B) Results of estimated ϕ_i^{12} under Scenario 2. (C) Results of estimated ϕ_i^{12} under Scenario 3. (D) Results of ϕ_i^{12} under Scenario 4.

2.2.2 Results under Scenarios 5-8

In this Section, we present simulation results for the full rank models with constraint "E^t" in Table 2 in the main paper, i.e. R-NE^{t}C, R-SE^{t}C and R-SE$^t_2$C. The boundary estimates of population sizes shown in Web Figure 7(A) is because we almost capture all of the individuals in the population during the study of length $K = 20$. The results in Web Figure 8 suggest that time-dependent transition probabilities obtained from the models incorporated with the constraint "E^t" are reliable due to near parameter redundancy as discussed in Section 1.3. Therefore, the use of constraint "E^t" is not recommended if we want to model time-dependent transition probabilities.

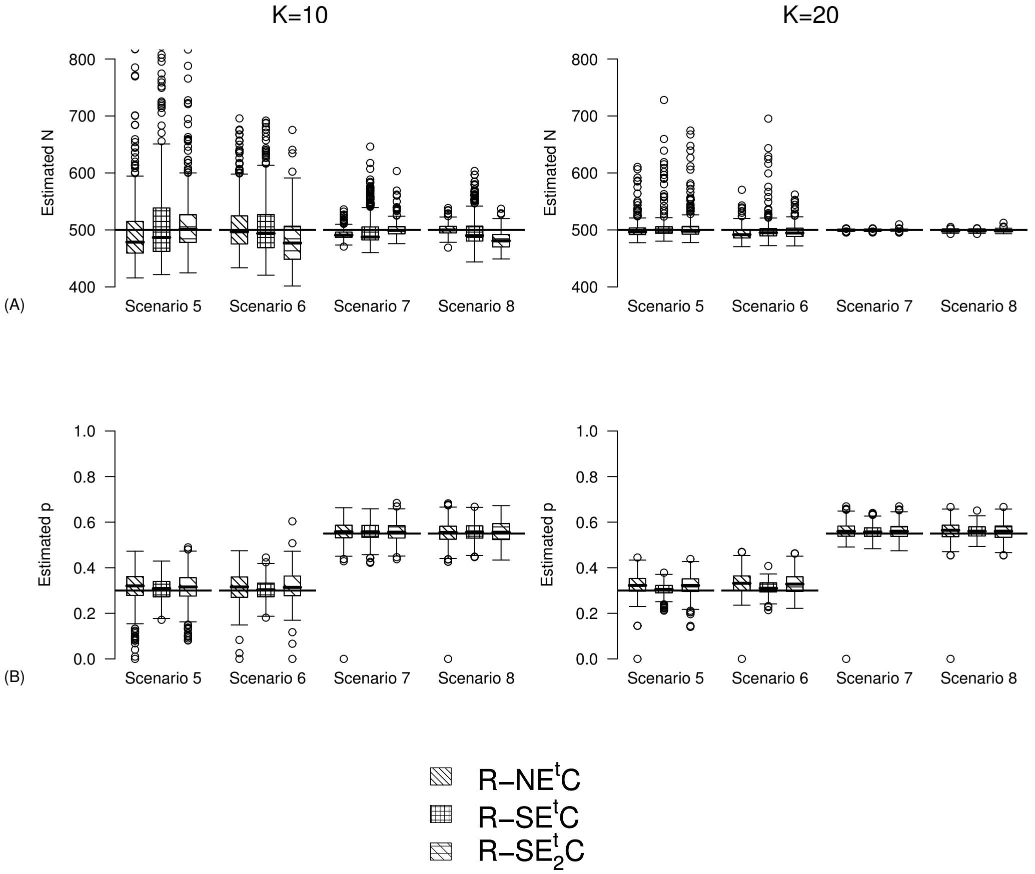

Web Figure 7: Results of estimated population sizes N (A) and capture probabilities p (B) for simulations with $K = 10$ and $K = 20$ sampling occasions displayed on the first four and last four columns respectively. The black horizontal lines are the true values used for simulating the data under different scenarios.

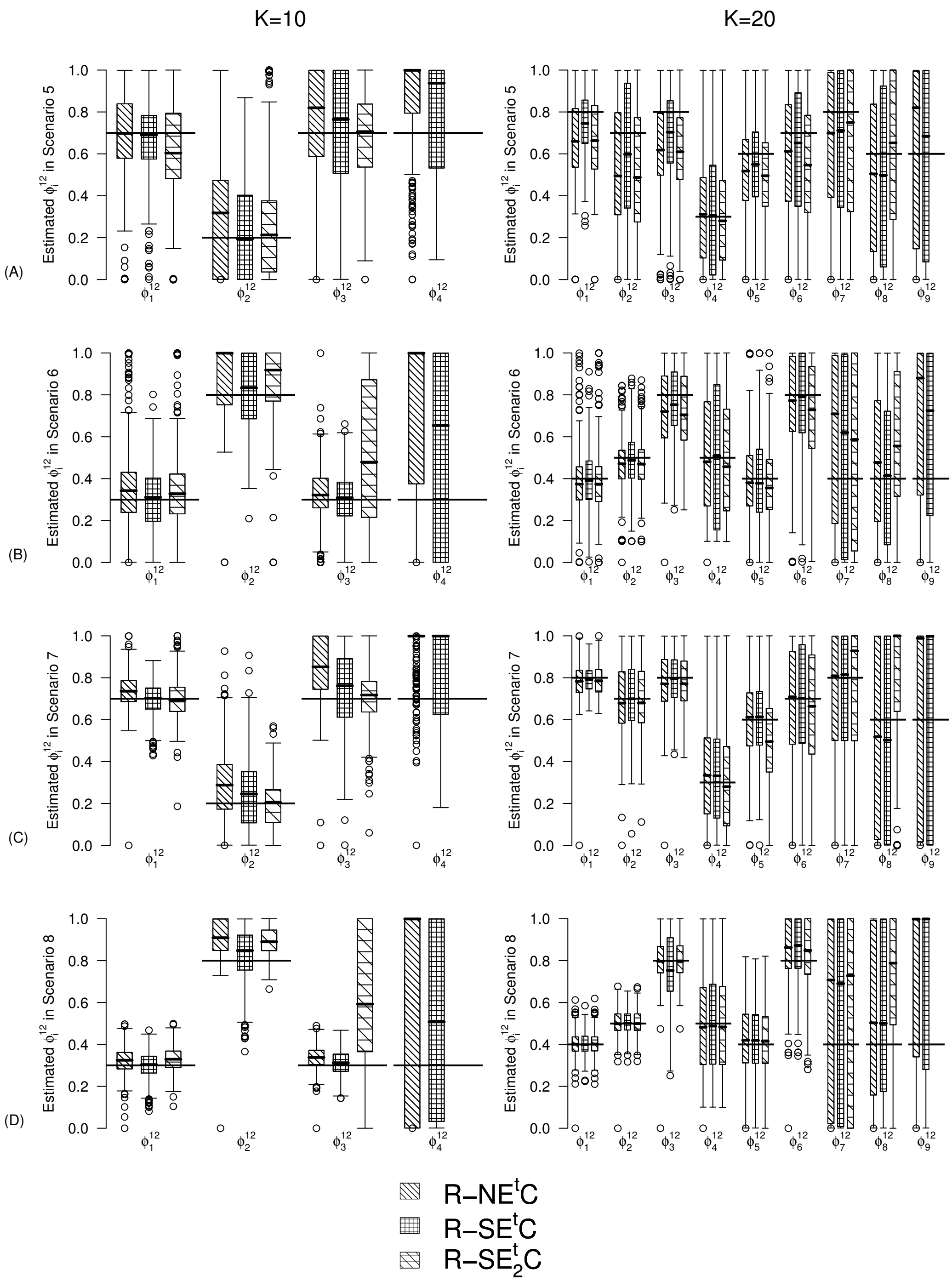

Web Figure 8: Results of estimated time-dependent transition probability ϕ_i^{12} for simulations with $K = 10$ and $K = 20$ sampling occasions displayed on the first four and last four columns respectively. The black horizontal lines are the true values used for simulating the data under different scenarios. (A) Results of estimated ϕ_i^{12} under Scenario 5. (B) Results of estimated ϕ_i^{12} under Scenario 6. (C) Results of estimated ϕ_i^{12} under Scenario 7. (D)Results of ϕ_i^{12} under Scenario 8.

2.2.3 Results for the GRM model

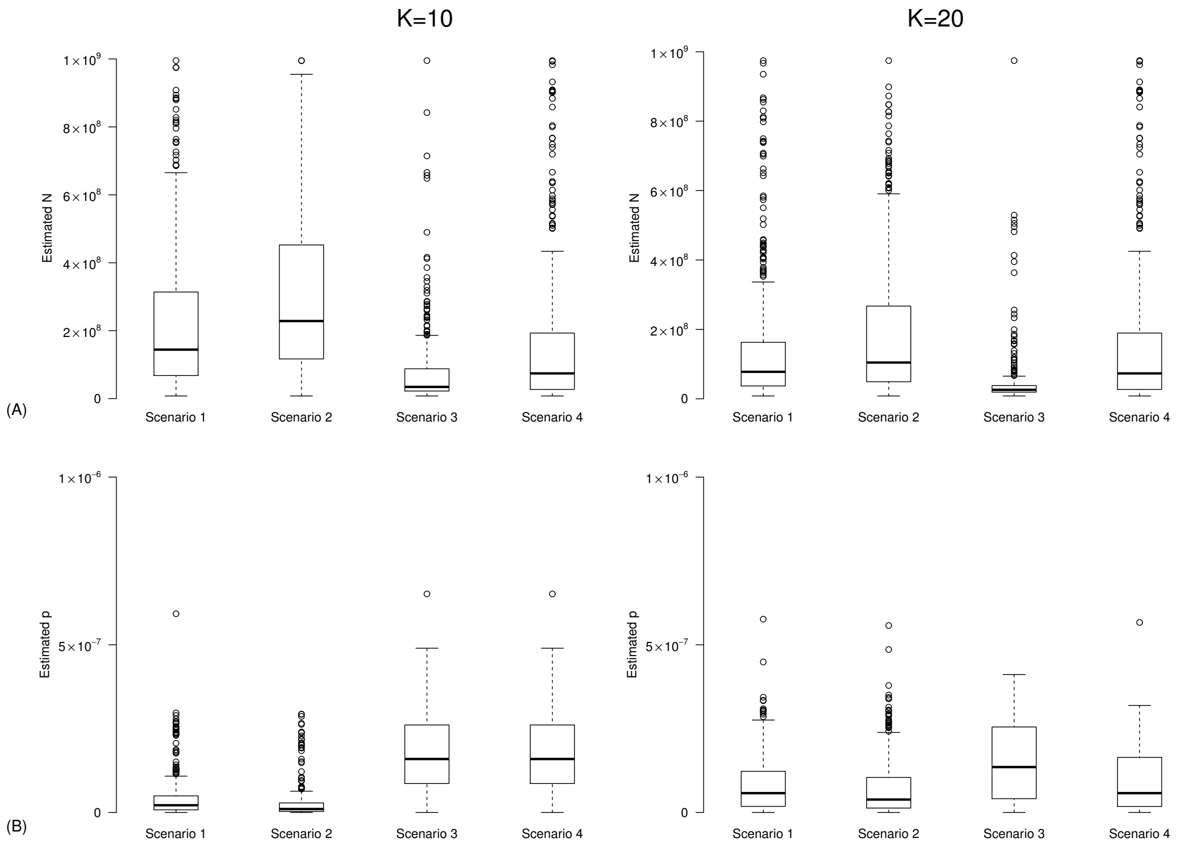

Web Figure 9: Results of estimated population sizes (A) and estimated capture probability p (B) for simulations with $K = 10$ and $K = 20$ sampling occasions displayed on the left and right column respectively. We fit the GRM models to the data simulated in Scenarios 1, 2, 3 and 4 under *Setting 4.2*. The true values for the capture probability is 0.3 under Scenarios 1 and 2, and 0.55 under Scenarios 3 and 4. In addition, the true value of the population size for simulating the data is $N = 500$.

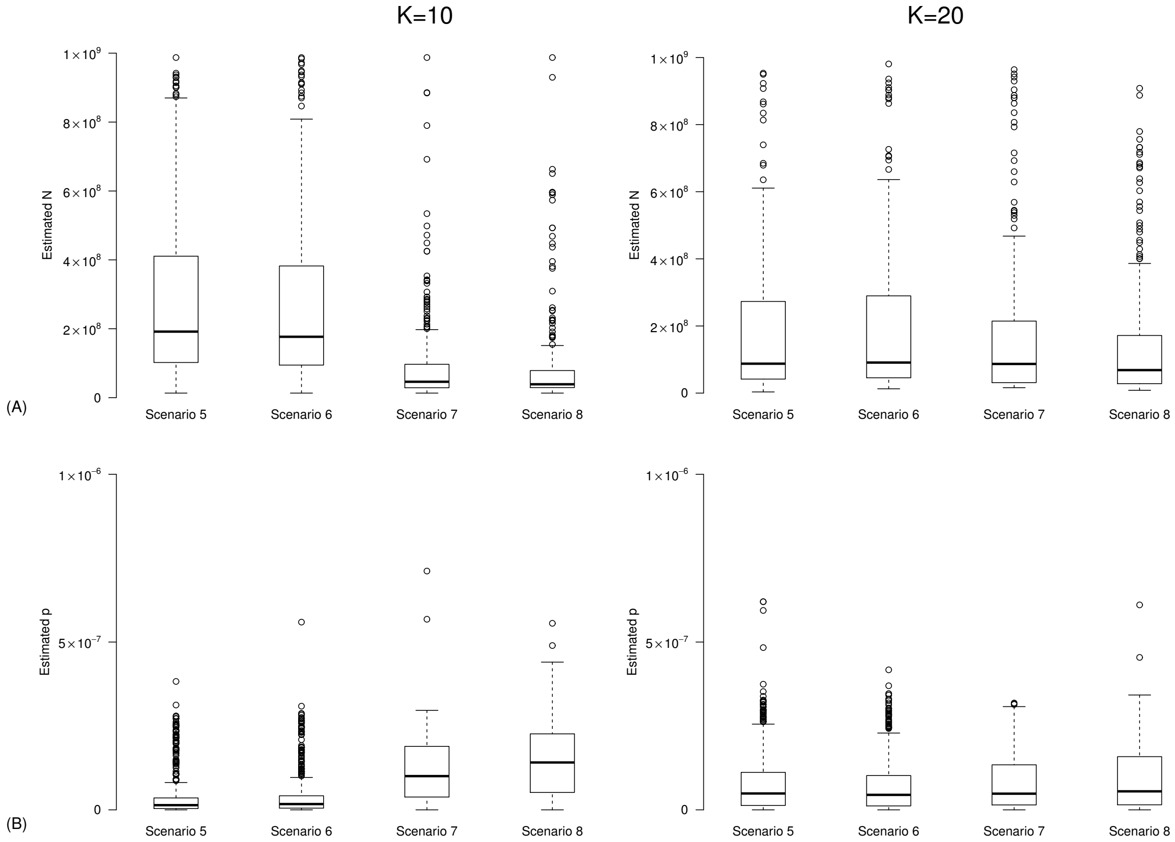

Web Figure 10: Results of estimated population sizes (A) and estimated capture probability p (B) for simulations with $K = 10$ and $K = 20$ sampling occasions displayed on the left and right column respectively. We fit the GRM models to the data simulated in Scenarios 5, 6, 7 and 8 under *Setting 4.2*. The true values for the capture probability is 0.3 under Scenarios 5 and 6, and 0.55 under Scenarios 7 and 8. In addition, the true value of the population size for simulating the data is $N = 500$.

2.3 Results under *Setting 4.3*

In this section, we present the simulation results under Scenarios 1-4 for the IRMER models in Table 2 in the paper. The detail for the simulation *Setting 4.3* is given in the main paper.

2.3.1 Results under Scenarios 1-4

We have demonstrated that we need to consider at least the combination of the constraint "R^t" and "S" in the model to get unbiased estimates of parameter in Section 2.2 as well as in the paper. In this section, we show that the integrated modelling approach (Besbeas et al., 2002) cannot improve the biased estimates in the IR-NR^tC model with the constraint "R^t" only as shown in Web Figure 11-13. In Web Figures 11 and 13, the estimated population sizes N, M and transition probabilities are biased low for the IR-NR^tC model across all Scenarios. Therefore, in the IRMER models the incorporation of both the constraint "R^t" and "S" is still needed.

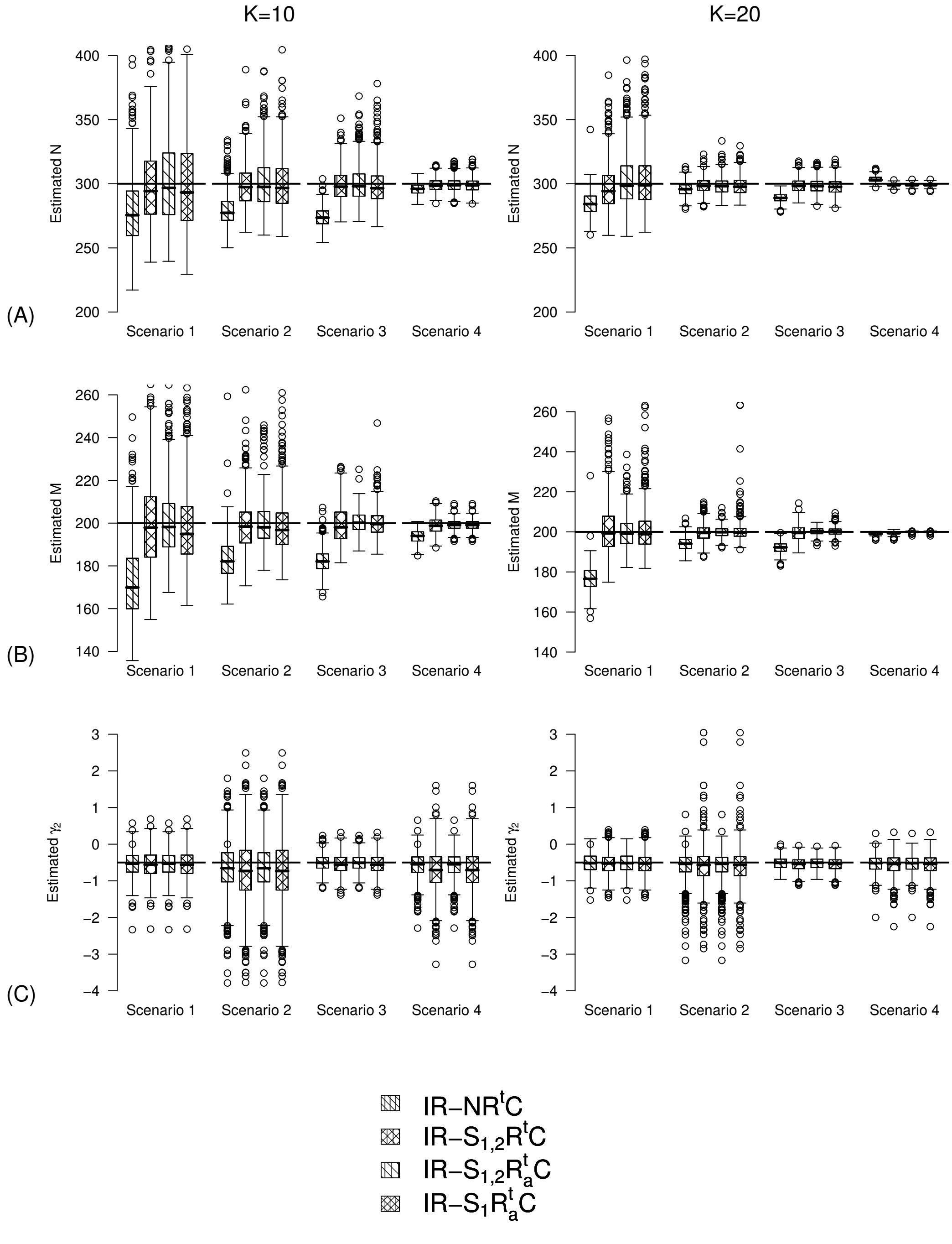

Web Figure 11: Results of estimated population sizes N and M for simulations under Scenarios 1, 2, 3 and 4 with $K = 10$ and $K = 20$ sampling occasions displayed on the first and second columns respectively. The black horizontal lines are the true values used for simulating the data under different scenarios. (A) Estimates of population size N. (B) Estimates of population size M. (C) Estimates of γ_2, which is an additive effect for transition probabilities for population 2 in the logistic regression.

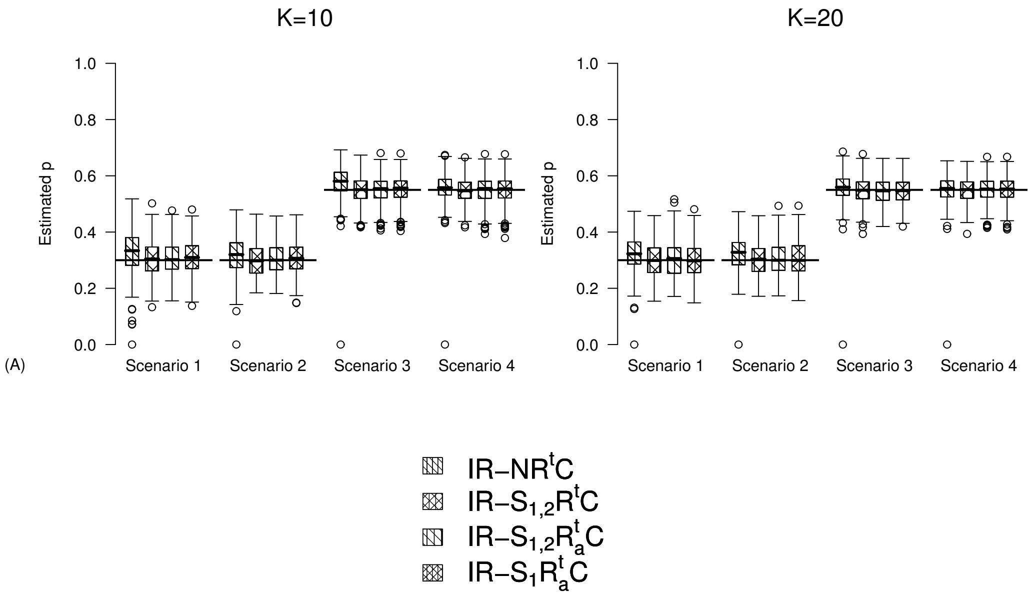

Web Figure 12: Results of estimated capture probability p for simulations with $K = 10$ and $K = 20$ sampling occasions displayed on the first four and last four columns respectively. The black horizontal lines are the true values used for simulating the data under different scenarios.

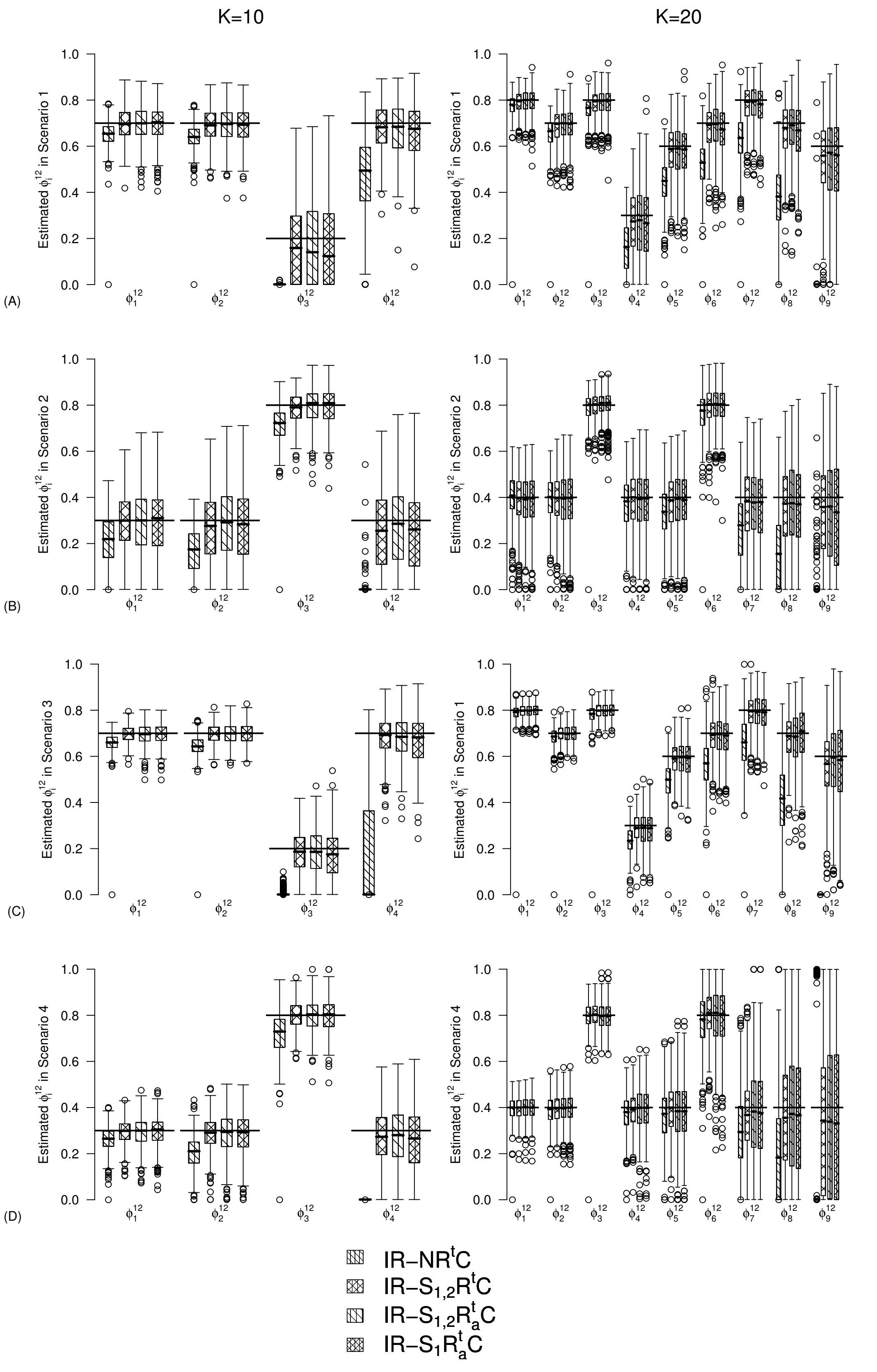

Web Figure 13: Results of estimated transition probabilities ϕ_i^{12} for simulations with $K = 10$ and $K = 20$ sampling occasions. The black horizontal lines are the true values used for simulating the data under different scenarios. (A) Estimates of transition probabilities ϕ_i^{12} under Scenario 1. (B) Estimates of transition probabilities ϕ_i^{12} under Scenario 2. (C) Estimates of transition probabilities ϕ_i^{12} under Scenario 3. (D) Estimates of transition probabilities ϕ_i^{12} under Scenario 4.

2.3.2 Results for the GRM model

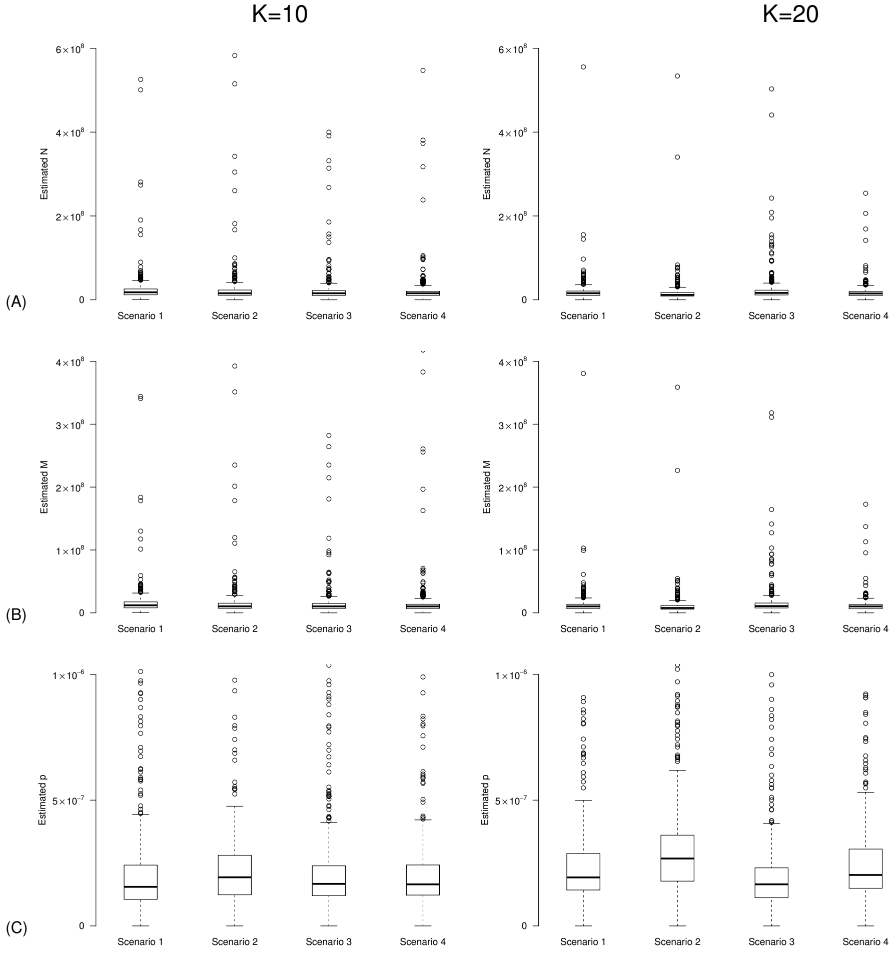

Web Figure 14: Results of estimated population sizes for the population 1 (A) and 2 (B), and estimated capture probability p (C) for simulations with $K = 10$ and $K = 20$ sampling occasions displayed on the left and right column respectively. The two populations in the geometric removal models (GRM) share the same constant capture probability. We fit the GRM models to the data simulated under *Setting 4.3*. The true values for the capture probability is 0.3 under Scenarios 1 and 2, and 0.55 under Scenarios 3 and 4. In addition, the true values of the population sizes for simulating the data are $N = 300$ and $M = 200$ for the population 1 and 2 respectively.

3 Web Appendix C: Investigation of the relaxation of constraint "R^{t}"

Here we investigate the generalization of the constraint "R^t" ($\phi_i^{12}+\phi_i^{21}=1$). We assume $\phi_i^{12}+\phi_i^{21}=v$, where $v \in [0,2]$ (denoted as "V^t" in Web Table 6). This constraint is motivated by the constraint of $\phi_i^{12}+\phi_i^{21}=1$, which relaxes the assumption of the sum of two transition probabilities at primary period i being equal to 1. The free parameters are fully time-dependent ϕ_i^{12} and v which is constant over time. Note that the limits of possible values of ϕ_i^{12} are conditional on v. The upper and lower bounds for ϕ_i^{12} are shown in the following,

$$\begin{cases} \phi_i^{12} \in [v-1,1], & \text{for} \quad v \in [1,2] \\ \phi_i^{12} \in [0,v], & \text{for} \quad v \in [0,1] \end{cases}$$

Parameter redundancy results are in Web Table 6. Simulations are displayed in Web Figures 19, 20 and 21 for $v = 0.4, 1,$ and 1.4 respectively. Unbiased estimates of capture probability are obtained for all possible values of v under all scenarios. We found that when $v > 1$, all of the estimates are unbiased under Scenario 2 and Scenario 4. However, the transition probabilities are overestimated under Scenario 1 and Scenario 3. None of the estimates is unbiased apart from the constant capture probability for other values of v.

If we regard v as a fixed number over time (not a free parameter), the parameter redundancy results are the same with those in Table 1 and 2 presented in the paper. Under this specification together with constraint "S", unbiased estimates can be obtained as shown in the simulation study for $v = 1$ in the paper.

Web Table 7: Results of deficiency of RMER models, where ϕ_i^{12} are time-varying over time. All models are diagnosed with an even number of sampling occasions, where there are two secondary samples within each primary period. h is the number of parameters in the model, r is the rank of the derivative matrix, d is the deficiency of the model, calculated as h-r. "t" denotes time-dependence, "c" denotes a constant parameter, and "t+cov" denotes additive effect in terms of covariates. PRS represents the parameter redundancy status, where FR indicates that all parameters in the model are theoretically estimable, PR indicates that the model is parameter redundant, NR indicates that the model is full rank but near parameter-redundant for the scenarios we considered. EP represents the estimable combinations of parameters if the model is parameter redundant. All results hold for $K \geq 4$ occasions.

Model code	Models	h	r	d	PRS	EP
R-NV^{t}C	$\pi, \phi^{12}(\text{t}), p(\text{c})$	K	K/2+1	K/2-1	PR	p,<br>π,<br>$f(p,\pi,\phi_1^{12},\phi_1^{21},v)$,<br>$\cdots$,<br>$f(p,\pi,\phi_1^{12},\cdots,\phi_{K/2-1}^{12},v)$
R-NV^{t}Z	$\pi, \phi^{12}(\text{t}), p(\text{t}+\text{cov})$	K+1	K/2+2	K/2-1	PR	α, β,<br>π,<br>$f(\alpha,\beta,\pi,\phi_1^{12},v)$,<br>$\cdots$,<br>$f(\alpha,\beta,\pi,\phi_1^{12},\cdots,\phi_{K/2-1}^{12},v)$
R-SV^{t}C	$\phi^{12}(\text{t}), p(\text{c})$	K/2	K/2	0	NR	-
R-SV^{t}Z	$\phi^{12}(\text{t}), p(\text{t}+\text{cov})$	K/2+1	K/2+1	0	NR	-

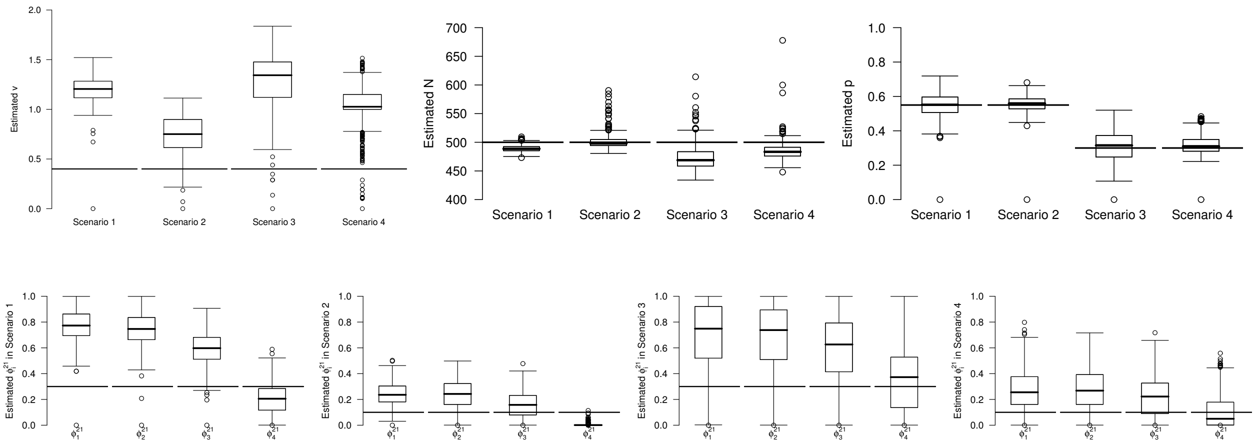

Web Figure 15: Simulation results of v, N, p and ϕ_i^{12} for R-NV^{t}C model with the constraint of $\phi_i^{12} + \phi_i^{21} = v$ where $v = 0.4$ when there are $K = 10$ sampling occasions with $T = 5$ primary periods and 2 secondary samples within each primary period. The true values of parameters are shown by the black horizontal lines.

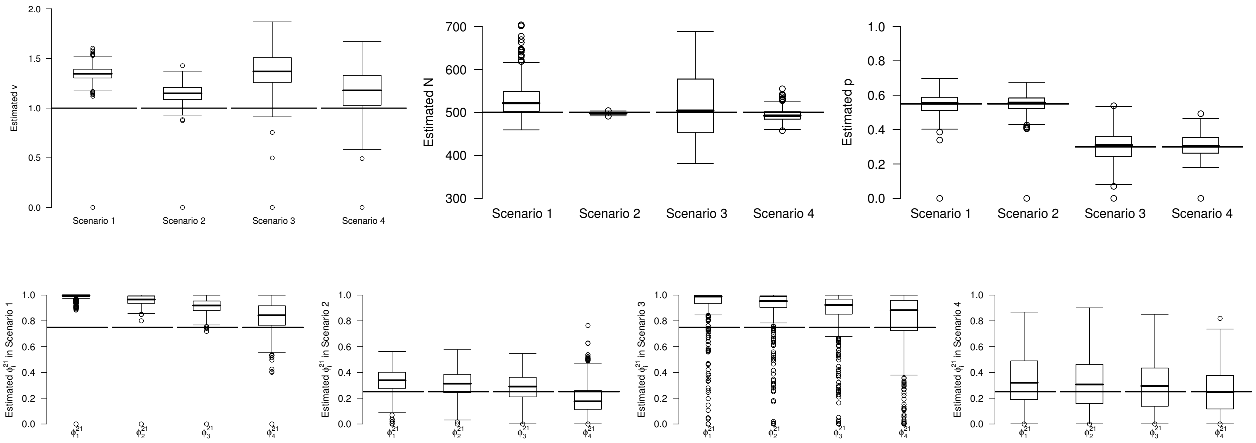

Web Figure 16: Simulation results of v, N, p and ϕ_i^{12} for R-NV^{t}C model with the constraint of $\phi_i^{12} + \phi_i^{21} = v$ where $v = 1$ when there are $K = 10$ sampling occasions with $T = 5$ primary periods and 2 secondary samples within each primary period. The true values of parameters are shown by the black horizontal lines.

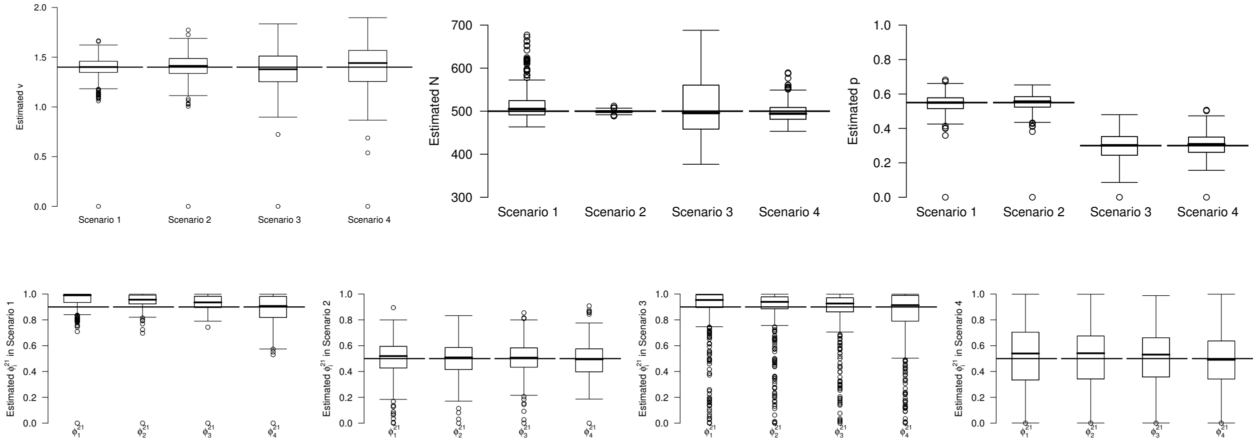

Web Figure 17: Simulation results of v, N, p and ϕ_i^{12} for R-NV^{t}C model with the constraint of $\phi_i^{12} + \phi_i^{21} = v$ where $v = 1.4$ when there are $K = 10$ sampling occasions with $T = 5$ primary periods and 2 secondary samples within each primary period. The true values of parameters are shown by the black horizontal lines.

4 Web Appendix D: Non-parametric bootstrap for removal data

In this section we describe the non-parametric bootstrap procedure used for analysis of the common lizard data presented in Section 5 of the paper. Bootstrap samples are obtained by sampling with replacement from the original data. Bootstrap estimates are denoted by $*$ on the superscript of the parameters, e.g. p^* denotes the bootstrap estimates of the constant capture probability. The algorithm is shown below.

- Step 1: rewrite the removal data in terms of individual removal records for all sampling occasions. If we removed n_k individuals at the kth sampling occasion, we rewrite this with n_k "k"s individual records. For example, the first three observations of the juvenile data set are $0, 5, 2$. We rewrite them as five "2"s and two "3"s, i.e. $2, 2, 2, 2, 2, 3, 3$ and repeat the same process for all of the observations.

- Step 2: sample D individual removal records with replacement and rewrite them in terms of removal count data by calculating the resulting frequency table, where D is equal to the total number of individuals we observed in the original data.

- Step 3: repeat Step 2 a large number of times (e.g. 500 resamples) to produce bootstrap samples.

- Step 4: fit the model to each bootstrap sample and obtain the bootstrap estimates.

- Step 5: Obtain the standard errors by calculating the standard deviation of the distribution of bootstrap estimates. The $(1 - a)\%$ confidence intervals are constructed by finding the $(a/2\%, 1-a/2\%)$ percentiles of the distribution of the bootstrap estimates. For example, the standard errors of p is calculated by the standard deviation of the distribution of p^* and the 95% confidence interval is computed by $(p^*_{0.025}, p^*_{0.975})$ where $p^*_{0.025}$ and $p^*_{0.975}$ are the 2.5% 97.5% percentiles of the bootstrapped p^* respectively.

In Web Figure 18 we show 500 bootstrap samples obtained for the juvenile and adult common lizard data sets analyzed in Section 5 of the paper. The model is fitted to each of the bootstrap samples and parameter estimates are computed that are subsequently used to construct confidence intervals and calculate standard errors.

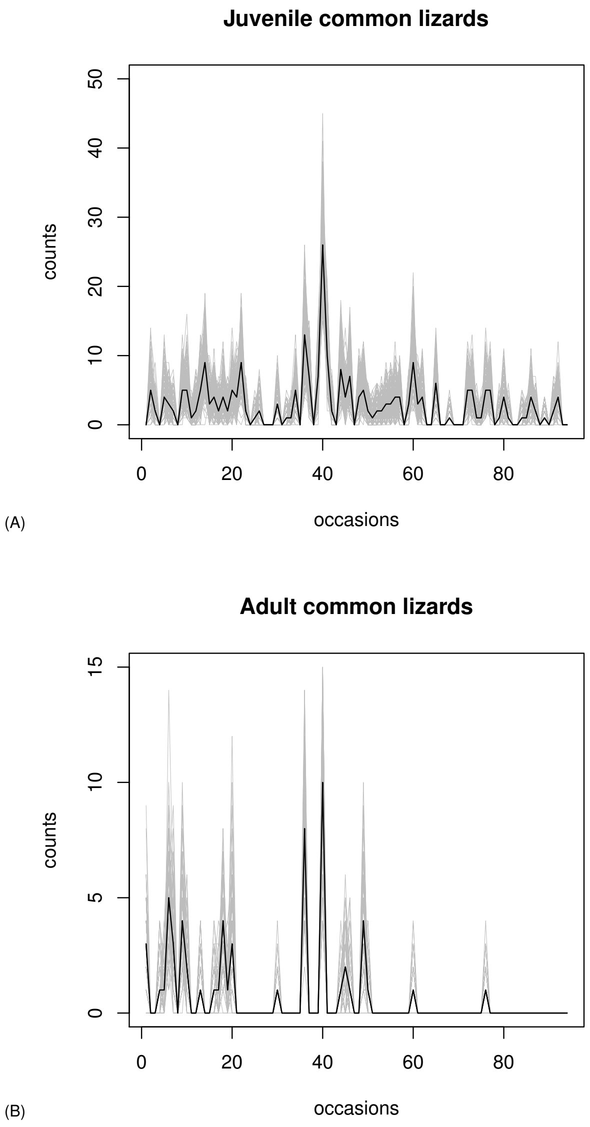

Web Figure 18: 500 bootstrap samples (gray lines) for juvenile (A) and adult (B) common lizard data (black lines).

5 Web Appendix E: Results for Data Analysis

In this section we present more results for the real data analyzed in Section 5 in the paper. In Web Table 8 We show all of the estimates apart from time-varying transition probabilities with standard errors and confidence interval calculated empirically from 500 bootstrap samples for the top three models in the Table 3 in the main paper. The fitted counts of individuals removed at each occasion for both juvenile and adult population are displayed in Web Figure 19. Furthermore, the estimates of time-dependent transition probabilities ϕ_i^{12} are shown in Web Figure 20.

As we described in Section 3. the constraint "V^t" ($\phi_i^{12} + \phi_i^{21} = v$, where $v \in [0, 2]$) only works when individuals have high mobility. Moreover, if we treat v as a fixed constant (denoted as "V^c") rather than a free parameter. The parameter redundancy results are the same with those with the constraint "R^t". We fit the model IR-S$_{\text{ju,ad}}$V$_a^c$Z to the real data over a grid fixed values of v. We show that the assumption of $\phi_i^{12} + \phi_i^{21} = 1$ results in the best fit for the common lizards data in Web Figure 21. Therefore we stick to the constraint "R" ($\phi_i^{12} + \phi_i^{21} = 1$) in our candidate models for the real data.

Web Table 8: Estimates and their standard errors (SE) and 95% confidence intervals (95% CI) for the three models with the lowest AIC in Table 3 in the paper for the common lizards data. $\hat{n}_{0,ju}$ and $\hat{n}_{0,ad}$ are the estimates of the number of juvenile and adult common lizards we failed to capture by the end of the study respectively. $\hat{\alpha}$ and $\hat{\beta}$ are the estimated logistic regression parameters (intercept and slope) for capture probability respectively. $\hat{\gamma_{ad}}$ is the estimate of additive effect for adult common lizards on transition probability. $\hat{\pi_{ad}}$ is the estimate of initial state parameter for the adult population.

Model	IR-S$_{\text{ju,ad}}$R$_a^t$Z	IR-S$_{\text{ju}}$R$_a^t$Z	IR-S$_{\text{ju,ad}}$R^{t}Z
ΔAIC	0	2.62	2.82
$\hat{n}_{0,ju}$	57.74	48.289	212.123
SE (95% CI)	146.02 (31.75, 474.33)	152.06 (31.23, 480.07)	100.93 (79.39, 458.75)
$\hat{n}_{0,ad}$	3.34	1.967	45.886
SE (95% CI)	49.76 (0.017, 180.11)	59.39 (0.04, 191.06)	28.87 (18.32, 110.09)
$\hat{\alpha}$	-1.98	-1.865	-3.028
SE (95% CI)	0.60 (-3.60, -1.46)	0.65 (-3.63 -1.47)	0.32 (-3.53, -2.32
$\hat{\beta}$	1.61	1.547	0.31
SE (95% CI)	0.37 (0.98, 2.32)	0.47 (-0.09, 2.41)	0.290 (1.37, 2.64)
$\hat{\gamma_{ad}}$	-0.97	-1.059	-
SE (95% CI)	1.525 (-1.84, 2.74)	1.57 (-1.87, 2.78)	-
$\hat{\pi_{ad}}$	-	0.373	-
SE (95% CI)	-	0.51 (0.51, 1.00)	-

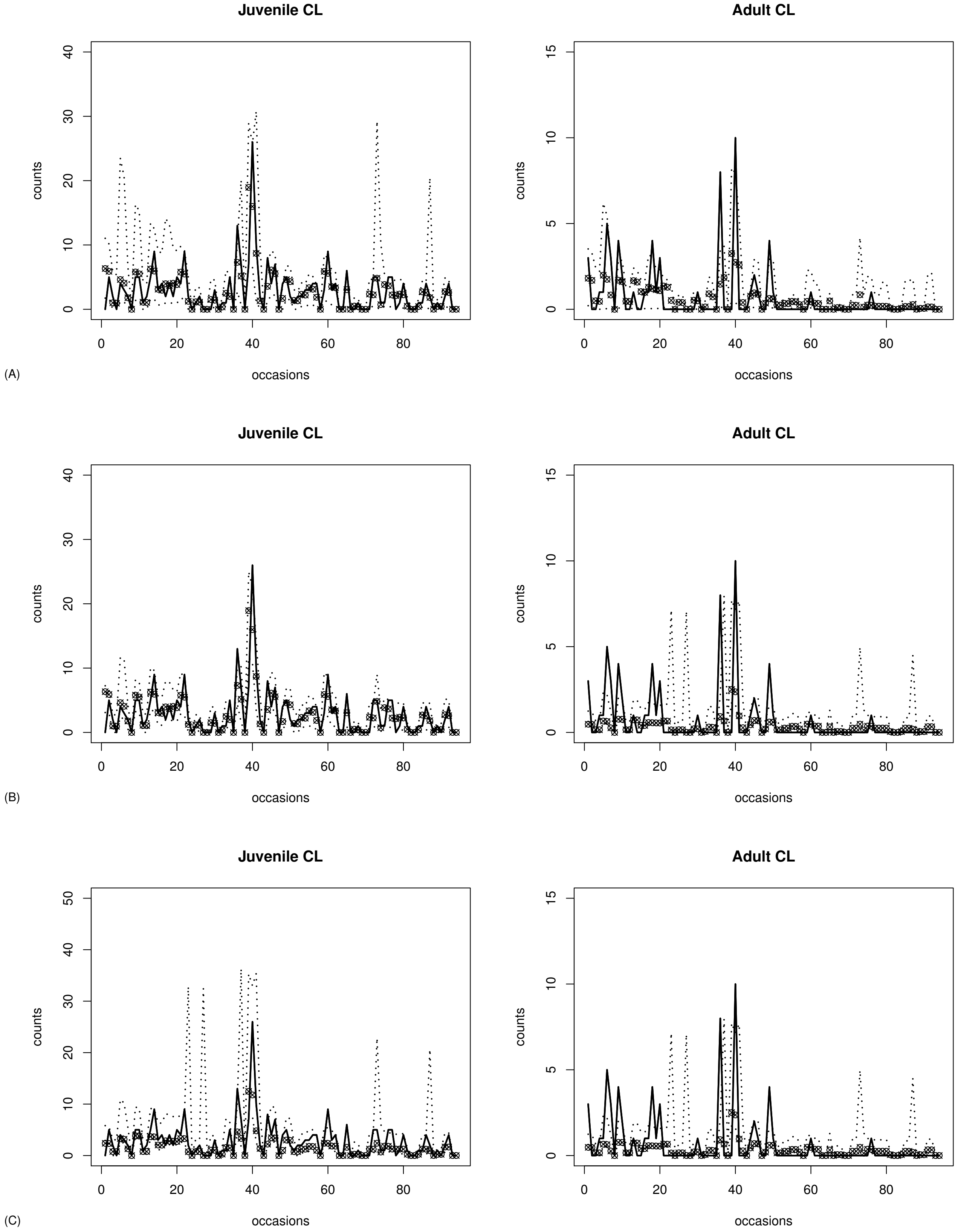

Web Figure 19: Results of predicted counts of common lizards (CL) removed at each occasion, shown by the circle and cross dots. Black solid lines are the real data. 95% confidence intervals obtained from 500 bootstrap samples are indicated within dotted lines. (A) Results obtained from the top model, IR-$\mathrm{S}_{\mathrm{ju,ad}}\mathrm{R}_a^t\mathrm{Z}$. (B) Results obtained from the IR-$\mathrm{S}_{\mathrm{ju}}\mathrm{R}_a^t\mathrm{Z}$ model with ΔAIC=2.62. (C) Results ordained from the IR-$\mathrm{S}_{\mathrm{ju,ad}}\mathrm{R}^t\mathrm{Z}$ model with ΔAIC=2.82.

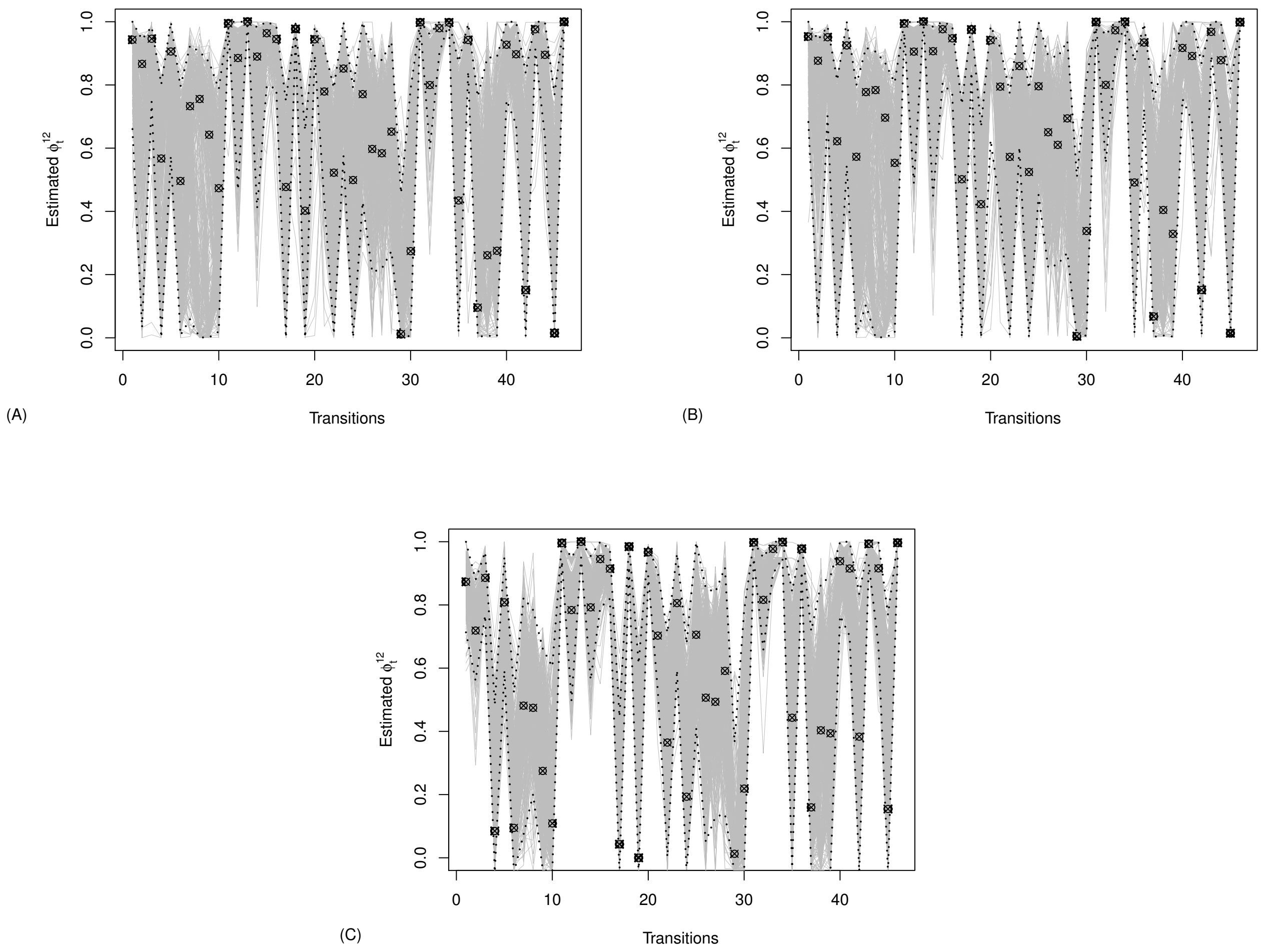

Web Figure 20: Results of estimates of transition probabilities ϕ_i^{12} obtained from the top three models for common lizards, shown by the circle and cross dots. Grey lines are the estimated ϕ_i^{12} from 500 bootstrap samples. 95% confidence intervals are indicated within dotted lines. (A) Estimates of ϕ_i^{12} obtained from the top model, , IR-$\mathrm{S}_{\mathrm{ju,ad}}\mathrm{R}_a^t\mathrm{Z}$. (B) Estimates of ϕ_i^{12} obtained from the second top model, IR-$\mathrm{S}_{\mathrm{ju}}\mathrm{R}_a^t\mathrm{Z}$ model with ΔAIC=2.62. (C) Estimates of ϕ_i^{12} ordained from the IR-$\mathrm{S}_{\mathrm{ju,ad}}\mathrm{R}^t\mathrm{Z}$ model with ΔAIC=2.82.

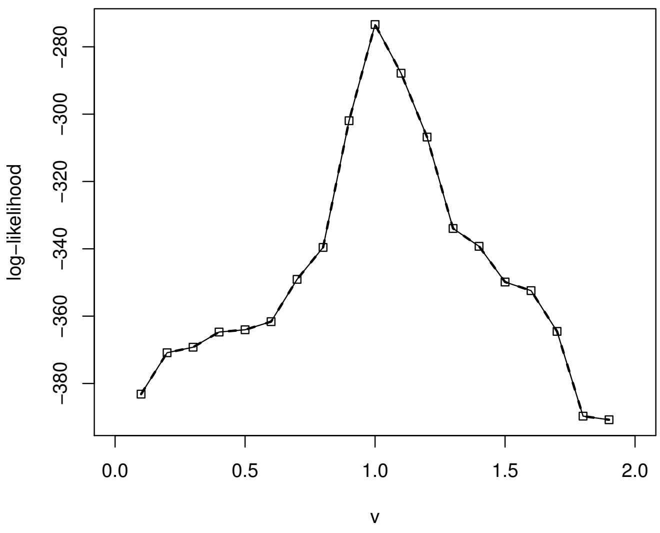

Web Figure 21: Results of log-likelihood values for different values of the sum of transition probabilities, where v ($\phi_i^{12} + \phi_i^{21} = v$, $v \in (0, 2)$) is a constant over time for common lizards data.

Web Table 9: Results of standardized smallest eigenvalues (S.Eig) and median relative bias in estimates $\hat{p}$ and $\hat{\psi}$ under different Scenarios, where true parameters are listed in p_{true} and ψ_{true}. All models are diagnosed with $K = 8$ sampling occasions where there are $T = 4$ primary periods and two secondary samples within each primary period. * indicates all individuals are assume to survive during the study.

Scenario	Model	p_{true}	ψ_{true}	S.Eig
1	R-NR^{t}C	0.3	*	2.02×10^{-2}
1	R-SR^{t}C	0.3	*	2.12×10^{-2}
A1	R-SR$_d^t$C	0.3	0.95	1.39×10^{-2}
A2	R-SR$_d^t$C	0.3	0.8	1.30×10^{-2}
A3	R-SR$_d^t$C	0.3	0.4	1.07×10^{-2}
3	R-NR^{t}C	0.55	*	1.12×10^{-1}
3	R-SR^{t}C	0.55	*	1.42×10^{-1}
B1	R-SR$_d^t$ C	0.55	0.95	1.14×10^{-1}
B2	R-SR$_d^t$C	0.55	0.8	1.10×10^{-1}
B3	R-SR$_d^t$C	0.55	0.4	6.05×10^{-2}

Web Table 10: Median relative bias (MBias) in estimates $\hat{p}$, $\hat{\psi}$ and $\hat{N}$ from the R-SR$_d^t$C model under different Scenarios. Simulations are conducted for simulated $N = 500$ individuals with $K = 20$ sampling occasions where there two secondary samples within each primary period. True values of true parameters of p_{true} and ψ_{true} are listed below. True transition probabilities used are the same (0.8,0.7,0.8,0.3,0.6,0.7,0.8,0.6,0.6) for every Scenario.

Scenario	p_{true}	ψ_{true}	$\hat{p}_{\text{MBias}}$	$\hat{\psi}_{\text{MBias}}$	$\hat{N}_{\text{MBias}}$
A1	0.3	0.95	5.53×10^{-3}	-1.41×10^{-1}	-2.04×10^{-1}
A2	0.3	0.8	2.14×10^{-1}	3.81×10^{-1}	-5.08×10^{-1}
A3	0.3	0.4	2.72×10^{-1}	5.52×10^{-1}	-3.92×10^{-1}
B1	0.55	0.95	7.03×10^{-3}	0.83×10^{-1}	-1.28×10^{-1}
B2	0.55	0.8	1.97×10^{-2}	6.73×10^{-2}	-3.78×10^{-1}
B3	0.55	0.4	7.64×10^{-2}	1.12×10^{0}	-6.40×10^{-1}

6 Web Appendix F: Consider mortality in the R-SR^{t}C model

In this section, we investigate whether we can estimate a constant survival probability ψ in the R-SR$_t$C model. We denote this new model by R-SR$_d^t$C as we consider an additional death state in the transition matrix, therefore there is a "d" in the subscript of R in the model notation.

This model R-SR$_d^t$C is determined to be full rank. We also investigate its eigenvalues under various Scenarios and we list the true values of parameters we used in Web Table 9. When capture probability is low its standardized smallest eigenvalue under Scenario A1, A2 or A3 is worse than those both R-NR^{t}C and R-SR^{t}C models under Scenario 1 as shown in Web Table 9. Similar conclusion is obtained when capture probability is high. Therefore it is not reliable to use as their standardized smallest eigenvalues are smaller than those from the R-NR^{t}C model which is a near parameter redundant model.

We also conducted simulation study for a population of $N = 500$ individuals with $K = 20$ sampling occasions. We list the true parameters in Web Table 10 where ϕ_i^{12} are (0.8,0.7,0.8,0.3,0.6,0.7,0.8,0.6,0.6) for each of the Scenarios considered, i.e. individuals are assume to tend to stay off-site. The estimated population sizes N are negatively biased across all scenarios in Web Table 10 and the estimates of survival probability $\hat{\psi}$ are over estimated apart from those under Scenario A1. The simulation results in Web Table 10 suggests the similar conclusion in Web Table 9, i.e. the R-SR$_d^t$C model with a constant survival probability cannot be used in practice.

References

Besbeas, P., Freeman, S. N., Morgan, B. J. T., and Catchpole, E. A. (2002). Integrating mark-recapture-recovery and census data to estimate animal abundance and demographic parameters. *Biometrics* **58,** 540–547.

Catchpole, E., Freeman, S. N., and Morgan, B. J. T. (1998). Estimation in parameter redundant models. *Biometrika* **85,** 462–468.

Catchpole, E. and Morgan, B. J. T. (1997). Detecting parameter redundancy. *Biometrics* **84,** 187–196.

Catchpole, E. A., Kgosi, P. M., and Morgan, B. J. T. (2001). On the near-singularity of models for animal recovery data. *Biometrics* **57,** 720–726.

Cole, D. J., Morgan, B. J. T., Catchpole, E. A., and Hubbard, B. A. (2012). Parameter redundancy in mark-recovery models. *Biometrical Journal* **54,** 507–523.

Cole, D. J., Morgan, B. J. T., and Titterington, D. M. (2010). The parametric structure of models. *Mathematical Biosciences* **228,** 16–30.
